# Supplementary material for: IL-12 and IL-23—Close Relatives with Structural Homologies but Distinct Immunological Functions
Source: Cells. 2020 Sep 28;9(10):2184. doi: 10.3390/cells9102184 (PMC7600943; doi:10.3390/cells9102184)
Supplement: Supplementary file 1 [file cells-09-02184-s001.pdf]

```
ATGAATCAGGTCACTATTCAATGGGATGCAGTAATAGCCCTTTACATACTCTTCAGCTGG 60
 M  N  Q  V  T  I  Q  W  D  A  V  I  A  L  Y  I  L  F  S  W  20
ACGGATCATGCC   AGT          GAGACATTA          TTTTGCGTACCCCTCGGCTAG
(T)(D)(H)(A)   (S)          (E)(T)(L)          (F)(C)(V)(P)(L)(G)(*)
 1  2  3  5     6            7  10 11           12 13 14 15 16 17 19
      CGG                   AAT                                AGATTG
      (R)                   (N)                                (R)(L)
       4                     8                                 18  20
                            GTT
                            (V)
                             9
```

01 - rs1408947770 - no publications, not in ClinVar - M1T - initiator codon variant
02 - rs746445795 - no publications, not in ClinVar - N2D
03 - rs1884444 - 43 publications, not in ClinVar - Q3H
04 - rs1884444 - no publications, not in ClinVar - 3QR
05 - rs111257711 - no publications, not in ClinVar - V4A
06 - rs770324228 - no publications, not in ClinVar - I6S
07 - rs144070297 - no publications, not in ClinVar - D9E
08 - rs775994483 - no publications, not in ClinVar - D9N
09 - rs1215777747 - no publications, not in ClinVar - D9V
10 - rs769230441 - no publications, not in ClinVar - A10T
11 - rs1256418489 - no publications, not in ClinVar - V11L
12 - rs1292287814 - no publications, not in ClinVar - L14F
13 - rs774581386 - no publications, not in ClinVar - Y15C
14 - rs761754539 - no publications, not in ClinVar - I16V
15 - rs1241516896 - no publications, not in ClinVar - L17P
16 - rs1353572541 - no publications, not in ClinVar - F18L
17 - rs185697788 - no publications, not in ClinVar - S19G
18 - rs760923055 - no publications, not in ClinVar - S19R
19 - rs765086515 - no publications, not in ClinVar - W20* - Stop Codon variant
20 - rs765086515 - no publications, not in ClinVar - W20L

#ROW_NO.	INPUT	PROTEIN_ID	POSITION	RESIDUE_REF	RESIDUE_ALT	SCORE	PREDICTION (cutoff=-2.5)	#SEQ	#CLUSTER	SCORE	PREDICTION (cutoff=0.05)	MEDIAN_INFO	#SEQ
1	ENSP00000321345,1,M,T	ENSP00000321345	1	M	T	-2.47	Neutral	146	30	0	Damaging	3.10	28
2	ENSP00000321345,2,N,D	ENSP00000321345	2	N	D	-1.04	Neutral	146	30	172	Tolerated	3.10	28
3	ENSP00000321345,3,Q,H	ENSP00000321345	3	Q	H	0.05	Neutral	146	30	381	Tolerated	3.10	28
4	ENSP00000321345,3,Q,R	ENSP00000321345	3	Q	R	-0.50	Neutral	146	30	379	Tolerated	3.10	28
5	ENSP00000321345,4,V,A	ENSP00000321345	4	V	A	-0.56	Neutral	146	30	174	Tolerated	3.04	29
6	ENSP00000321345,6,I,S	ENSP00000321345	6	I	S	-0.54	Neutral	146	30	386	Tolerated	3.01	31
7	ENSP00000321345,9,D,E	ENSP00000321345	9	D	E	0.19	Neutral	146	30	721	Tolerated	2.91	40
8	ENSP00000321345,9,D,N	ENSP00000321345	9	D	N	-0.30	Neutral	146	30	145	Tolerated	2.91	40
9	ENSP00000321345,9,D,V	ENSP00000321345	9	D	V	-0.99	Neutral	146	30	66	Tolerated	2.91	40
10	ENSP00000321345,10,A,T	ENSP00000321345	10	A	T	-0.09	Neutral	146	30	77	Tolerated	2.91	41
11	ENSP00000321345,11,V,L	ENSP00000321345	11	V	L	-0.33	Neutral	146	30	123	Tolerated	2.89	44
12	ENSP00000321345,14,L,F	ENSP00000321345	14	L	F	-1.55	Neutral	146	30	145	Tolerated	2.86	53
13	ENSP00000321345,15,Y,C	ENSP00000321345	15	Y	C	-1.21	Neutral	146	30	188	Tolerated	2.86	53
14	ENSP00000321345,16,I,V	ENSP00000321345	16	I	V	-0.12	Neutral	146	30	359	Tolerated	2.86	53
15	ENSP00000321345,17,L,P	ENSP00000321345	17	L	P	-3.17	Deleterious	146	30	3	Damaging	2.86	53
16	ENSP00000321345,18,F,L	ENSP00000321345	18	F	L	-0.98	Neutral	146	30	294	Tolerated	2.86	53
17	ENSP00000321345,19,S,G	ENSP00000321345	19	S	G	-1.19	Neutral	146	30	496	Tolerated	2.86	53
18	ENSP00000321345,19,S,R	ENSP00000321345	19	S	R	-0.22	Neutral	146	30	551	Tolerated	2.86	53
19	ENSP00000321345,20,W,L	ENSP00000321345	20	W	L	-2.42	Neutral	146	30	74	Tolerated	2.86	53

http://provean.jcvi.org/index.php

TGT**CATGGA**GGAATT**ACAAATATA**AACTGCTCTGGCCAC**ATCTGG**GTA**GAA**CCA**GCC**ACA 120
C H G G I T N I N C S G H I W V E P A T 40

TATAGA **GCAAGTAAA** **GTCTGT** **GAC** **ACC**
(Y) (R) **(A) (S) (K)** **(V) (C)** **(D)** **(T)**
21 22 23 24 25 26 27 28 29

21 - rs1474792230 – no publications, not in ClinVar – H22Y
22 - rs752478137 – no publications, not in ClinVar – G23R

---

23 - rs1290144534 – no publications, not in ClinVar – T26A
24 - rs1465641771 – no publications, not in ClinVar – N27S Bloch et al.
25 - rs1162714374 – no publications, not in ClinVar – I28K Bloch et al.
26 - rs1156783916 – no publications, not in ClinVar – I34V
27 - rs1396700760 – no publications, not in ClinVar – W35C
28 - rs762697123 – no publications, not in ClinVar – E37D
29 - rs1570774918 – no publications, not in ClinVar – A39T

#ROW_NO.	INPUT	PROTEIN_ID	POSITION	RESIDUE_REF	RESIDUE_ALT	SCORE	PREDICTION (cutoff=-2.5)	#SEQ	#CLUSTER	SCORE	PREDICTION (cutoff=0.05)	MEDIAN_INFO	#SEQ
1	ENSP00000321345,22,H,Y	ENSP00000321345	22	H	Y	-0.74	Neutral	146	30	238	Tolerated	2.84	55
2	ENSP00000321345,23,G,R	ENSP00000321345	23	G	R	-1.84	Neutral	146	30	5	Damaging	2.84	55
3	ENSP00000321345,26,T,A	ENSP00000321345	26	T	A	-0.43	Neutral	146	30	194	Tolerated	2.89	46
4	ENSP00000321345,27,N,S	ENSP00000321345	27	N	S	-0.11	Neutral	146	30	841	Tolerated	2.91	45
5	ENSP00000321345,28,I,K	ENSP00000321345	28	I	K	-1.50	Neutral	146	30	2	Damaging	2.88	54
6	ENSP00000321345,34,I,V	ENSP00000321345	34	I	V	0.03	Neutral	146	30	994	Tolerated	2.80	79
7	ENSP00000321345,35,W,C	ENSP00000321345	35	W	C	-2.94	Deleterious	146	30	14	Damaging	2.84	76
8	ENSP00000321345,37,E,D	ENSP00000321345	37	E	D	-1.09	Neutral	146	30	11	Damaging	2.81	82
9	ENSP00000321345,39,A,T	ENSP00000321345	39	A	T	-1.70	Neutral	146	30	6	Damaging	2.81	82

ATTTTTAAG**ATG**GGTATG**AAT**ATC**TCTATATATTGC**CAAGCA**GCAATTAAG**AACTGCCAA 180
I F K M G M N I S I Y C Q A A I K N C Q 60

ACG (T) 30 — GTG (V) 31 — AGT (S) 32 — TATATGCATTTC (Y) (M) (H) (F) 33 34 36 37 — GTA (V) 35 — GAATTTGAG (E) (F) (E) 38 40 41 — ACA (T) 39

30 - rs144606217 – no publications, not in ClinVar – M44T
31 - rs763854576 – no publications, not in ClinVar – M44V
32 - rs761804830 – no publications, not in ClinVar – N47S
33 - rs1356970161 – no publications, not in ClinVar – S49Y
34 - rs749888878 – no publications, not in ClinVar – I50M
35 - rs766991367 – no publications, not in ClinVar – I50V
36 - rs1011510628 – no publications, not in ClinVar – Y51H
37 – rs1260672434 – no publications, not in ClinVar – C52F
38 - rs201210897 – no publications, not in ClinVar – A55E
39 - rs539781624 – no publications, not in ClinVar – A55T
40 - rs767092630 – no publications, not in ClinVar – I56F
41 - rs779308889 – no publications, not in ClinVar – K57E

#ROW_NO.	INPUT	PROTEIN_ID	POSITION	RESIDUE_REF	RESIDUE_ALT	SCORE	PREDICTION (cutoff=-2.5)	#SEQ	#CLUSTER	SCORE	PREDICTION (cutoff=0.05)	MEDIAN_INFO	#SEQ
1	ENSP00000321345,44,M,T	ENSP00000321345	44	M	T	-3.10	Deleterious	146	30	3	Damaging	2.81	82
2	ENSP00000321345,44,M,V	ENSP00000321345	44	M	V	-1.67	Neutral	146	30	60	Tolerated	2.81	82
3	ENSP00000321345,47,N,S	ENSP00000321345	47	N	S	-1.45	Neutral	146	30	152	Tolerated	2.84	80
4	ENSP00000321345,49,S,Y	ENSP00000321345	49	S	Y	-3.41	Deleterious	146	30	1	Damaging	2.81	82
5	ENSP00000321345,50,I,M	ENSP00000321345	50	I	M	-1.67	Neutral	146	30	2	Damaging	2.81	82
6	ENSP00000321345,50,I,V	ENSP00000321345	50	I	V	-0.03	Neutral	146	30	303	Tolerated	2.81	82
7	ENSP00000321345,51,Y,H	ENSP00000321345	51	Y	H	-1.95	Neutral	146	30	16	Damaging	2.81	82
8	ENSP00000321345,52,C,F	ENSP00000321345	52	C	F	-8.60	Deleterious	146	30	1	Damaging	2.81	82
9	ENSP00000321345,55,A,E	ENSP00000321345	55	A	E	-0.29	Neutral	146	30	321	Tolerated	2.82	80
10	ENSP00000321345,55,A,T	ENSP00000321345	55	A	T	-0.10	Neutral	146	30	233	Tolerated	2.82	80
11	ENSP00000321345,56,I,F	ENSP00000321345	56	I	F	-1.12	Neutral	146	30	226	Tolerated	2.84	80
12	ENSP00000321345,57,K,E	ENSP00000321345	57	K	E	-0.81	Neutral	146	30	85	Tolerated	2.81	81

```
CCAAGGAAACTTCATTTTTATAAAAATGGCATCAAAGAAAGATTTCAAATCACAAGGATT 240
 P  R  K  L  H  F  Y  K  N  G  I  K  E  R  F  Q  I  T  R  I  80
TCA    GAAGTTAAT                    GTCGTC        ATATAT    ACCGCA
(S)     (E) (V) (N)                  (V) (V)       (I) (Y)   (T) (A)
 42      44  45  47                   48  49        51  52    53  54
CTA       CCT                          TTC
(L)       (P)                          (F)
 43        46                           50
```

42 - rs1363189594 – no publications, not in ClinVar – P61S
43 - rs1466428054 – no publications, not in ClinVar – P61L
44 - rs1469323792 – no publications, not in ClinVar – K63E
45 - rs747027115 – no publications, not in ClinVar – L64V
46 - rs771174962 – no publications, not in ClinVar – L64P
47 - rs776785412 – no publications, not in ClinVar – H65N
48 - rs759723554 – no publications, not in ClinVar – G70V
49 - rs576532413 – no publications, not in ClinVar – I71V
50 - rs576532413 – no publications, not in ClinVar – I71F
51 - rs1281925695 – no publications, not in ClinVar – R74I
52 - rs1320853001 – no publications, not in ClinVar – F75Y
53 - rs1202465423 – no publications, not in ClinVar – I77T
54 - rs558208409 – no publications, not in ClinVar – T78A

#ROW_NO.	INPUT	PROTEIN_ID	POSITION	RESIDUE_REF	RESIDUE_ALT	SCORE	PREDICTION (cutoff=-2.5)	#SEQ	#CLUSTER	SCORE	PREDICTION (cutoff=0.05)	MEDIAN_INFO	#SEQ
1	ENSP00000321345,61,P,S	ENSP00000321345	61	P	S	-1.78	Neutral	146	30	71	Tolerated	2.82	78
2	ENSP00000321345,61,P,L	ENSP00000321345	61	P	L	-2.78	Deleterious	146	30	89	Tolerated	2.82	78
3	ENSP00000321345,63,K,E	ENSP00000321345	63	K	E	-1.21	Neutral	146	30	290	Tolerated	2.82	78
4	ENSP00000321345,64,L,V	ENSP00000321345	64	L	V	-0.65	Neutral	146	30	113	Tolerated	2.83	77
5	ENSP00000321345,64,L,P	ENSP00000321345	64	L	P	-3.12	Deleterious	146	30	4	Damaging	2.83	77
6	ENSP00000321345,65,H,N	ENSP00000321345	65	H	N	-1.48	Neutral	146	30	233	Tolerated	2.81	81
7	ENSP00000321345,70,G,V	ENSP00000321345	70	G	V	-2.24	Neutral	146	30	45	Damaging	2.85	75
8	ENSP00000321345,71,I,V	ENSP00000321345	71	I	V	0.05	Neutral	146	30	520	Tolerated	2.88	78
9	ENSP00000321345,71,I,F	ENSP00000321345	71	I	F	-0.38	Neutral	146	30	448	Tolerated	2.88	78
10	ENSP00000321345,74,R,I	ENSP00000321345	74	R	I	-2.30	Neutral	146	30	3	Damaging	2.84	79
11	ENSP00000321345,75,F,Y	ENSP00000321345	75	F	Y	-0.05	Neutral	146	30	233	Tolerated	2.84	79
12	ENSP00000321345,77,I,T	ENSP00000321345	77	I	T	-0.53	Neutral	146	30	298	Tolerated	2.82	114
13	ENSP00000321345,78,T,A	ENSP00000321345	78	T	A	-1.17	Neutral	146	30	79	Tolerated	2.82	111

```
AATAAAACAACAGCTCGGCTTTGGTATAAAAACTTTCTGGAACCACATGCTTCTATGTAC 300
 N  K  T  T  A  R  L  W  Y  K  N  F  L  E  P  H  A  S  M  Y  100
AAA      ---ACTCAG   TTGTGT   GAC            CGT      GTG
(K)         (T)(Q)   (L)(C)   (D)            (R)      (V)
 55      56  57 58    61 62    63             64       66
               CCG                           TAT
               (P)                           (Y)
                59                            65
               TGG
               (W)
                60
```

55 - rs1035929222 – no publications, not in ClinVar – N81K
56 - rs748507585 – no publications, not in ClinVar - T84del - in-frame deletion
57 - rs1433548222 – no publications, not in ClinVar – A85T
58 - rs76575803 – 1 publications, not in ClinVar - R86Q
58 - rs76575803 – no publications, not in ClinVar - R86P
60 - rs749929021 – no publications, not in ClinVar - R86W
61 - rs1171424420 – no publications, not in ClinVar – W88L
62 - rs1047010535 – no publications, not in ClinVar – Y89C
63 - rs1356496625 – no publications, not in ClinVar - N91D
64 - rs753050122 – no publications, not in ClinVar – H96R
65 - rs906756964 – no publications, not in ClinVar - H96Y
66 - rs758835954 – no publications, not in ClinVar – M99V

#ROW_NO.	INPUT	PROTEIN_ID	POSITION	RESIDUE_REF	RESIDUE_ALT	SCORE	PREDICTION (cutoff=-2.5)	#SEQ	#CLUSTER	SCORE	PREDICTION (cutoff=0.05)	MEDIAN_INFO	#SEQ
1	ENSP00000321345,81,N,K	ENSP00000321345	81	N	K	-3.89	Deleterious	146	30	3	Damaging	2.81	114
2	ENSP00000321345,84,T,	input format error											
3	ENSP00000321345,85,A,T	ENSP00000321345	85	A	T	-0.90	Neutral	146	30	124	Tolerated	2.81	111
4	ENSP00000321345,86,R,Q	ENSP00000321345	86	R	Q	0.12	Neutral	146	30	411	Tolerated	2.80	112
5	ENSP00000321345,86,R,P	ENSP00000321345	86	R	P	-1.12	Neutral	146	30	56	Tolerated	2.80	112
6	ENSP00000321345,88,W,L	ENSP00000321345	88	W	L	-1.07	Neutral	146	30	641	Tolerated	2.81	108
7	ENSP00000321345,89,Y,C	ENSP00000321345	89	Y	C	-2.12	Neutral	146	30	8	Damaging	2.81	108
8	ENSP00000321345,91,N,D	ENSP00000321345	91	N	D	-1.19	Neutral	146	30	348	Tolerated	2.81	113
9	ENSP00000321345,96,H,R	ENSP00000321345	96	H	R	-0.34	Neutral	146	30	627	Tolerated	2.82	95
10	ENSP00000321345,96,H,Y	ENSP00000321345	96	H	Y	-1.53	Neutral	146	30	287	Tolerated	2.82	95
11	ENSP00000321345,99,M,V	ENSP00000321345	99	M	V	-0.03	Neutral	146	30	1.000	Tolerated	2.80	115

```
TGCACTGCTGAATGTCCCAAACATTTTCAAGAGACACTGATATGTGGAAAAGACATTTCT 360
 C  T  A  E  C  P  K  H  F  Q  E  T  L  I  C  G  K  D  I  S  120
TACCCT         TGGTCCATATAT            AAG            AGT
(Y) (P)        (W) (S) (I) (Y)         (K)            (S)
 67  68         70  71  72  73          74             75
    GCT                                               CGT
    (A)                                               (R)
     69                                                76
                                                      GGT
                                                      (G)
                                                       77
```

67 - rs1301889923 – no publications, not in ClinVar – C101Y
68 - rs1345386569 – no publications, not in ClinVar - T102P Bloch et al.
69 - rs1345386569 – no publications, not in ClinVar - T102A Bloch et al.
70 - rs1295595033 – no publications, not in ClinVar – C105W
71 - rs747115036 – no publications, not in ClinVar – P106S
72 - rs1368052402 – no publications, not in ClinVar - K107I Bloch et al.
73 - rs757367045 – no publications, not in ClinVar - H108Y Bloch et al.
74 - rs1250378988 – no publications, not in ClinVar – E111K
75 - rs746144971 – no publications, not in ClinVar – C115S
76 - rs746144971 – no publications, not in ClinVar – C115R
77 - rs746144971 – no publications, not in ClinVar – C115G

#ROW_NO.	INPUT	PROTEIN_ID	POSITION	RESIDUE_REF	RESIDUE_ALT	SCORE	PREDICTION (cutoff=-2.5)	#SEQ	#CLUSTER	SCORE	PREDICTION (cutoff=0.05)	MEDIAN_INFO	#SEQ
1	ENSP00000321345,101,C,Y	ENSP00000321345	101	C	Y	-7.88	Deleterious	146	30	1	Damaging	2.80	116
2	ENSP00000321345,102,T,P	ENSP00000321345	102	T	P	-2.19	Neutral	146	30	7	Damaging	2.80	116
3	ENSP00000321345,102,T,A	ENSP00000321345	102	T	A	-1.66	Neutral	146	30	14	Damaging	2.80	116
4	ENSP00000321345,105,C,W	ENSP00000321345	105	C	W	-5.62	Deleterious	146	30	0	Damaging	2.80	116
5	ENSP00000321345,106,P,S	ENSP00000321345	106	P	S	-0.24	Neutral	146	30	467	Tolerated	2.83	98
6	ENSP00000321345,107,K,I	ENSP00000321345	107	K	I	-1.40	Neutral	146	30	75	Tolerated	2.82	114
7	ENSP00000321345,108,H,Y	ENSP00000321345	108	H	Y	-1.09	Neutral	146	30	588	Tolerated	2.84	114
8	ENSP00000321345,111,E,K	ENSP00000321345	111	E	K	-1.47	Neutral	146	30	69	Tolerated	2.80	117
9	ENSP00000321345,115,C,S	ENSP00000321345	115	C	S	-6.00	Deleterious	146	30	42	Damaging	2.81	115
10	ENSP00000321345,115,C,R	ENSP00000321345	115	C	R	-7.40	Deleterious	146	30	2	Damaging	2.81	115
11	ENSP00000321345,115,C,G	ENSP00000321345	115	C	G	-7.22	Deleterious	146	30	14	Damaging	2.81	115

```
TCTGGATATCCGCCAGATATTCCTGATGAAGTAACCTGTGTCATTTATGAATATTCAGGC 420
 S  G  Y  P  P  D  I  P  D  E  V  T  C  V  I  Y  E  Y  S  G  140
           CTG   AAT       GAA    GCAAAT       TTT
           (L)   (N)       (E)    (A) (N)      (F)
           78    79        83     84  85       87
                 CAT              ATT          ACT
                 (H)              (I)          (T)
                 80               86           88
                 TAT
                 (Y)
                 81
                 GGT
                 (G)
                 82
```

```
78 - rs781262110     - no publications, not in ClinVar - P124L
79 - rs780271790     - no publications, not in ClinVar - D126N
80 - rs780271790     - no publications, not in ClinVar - D126H
81 - rs780271790     - no publications, not in ClinVar - D126Y
82 - rs1162079771    - no publications, not in ClinVar - D126G
83 - rs539497366     - no publications, not in ClinVar - D129E
84 - rs771938488     - no publications, not in ClinVar - V131A
85 - rs773141807     - no publications, not in ClinVar - T132N
86 - rs773141807     - no publications, not in ClinVar - T132I
87 - rs192128821     - no publications, not in ClinVar - I135F
88 - rs201136288     - no publications, not in ClinVar - I135T
```

#ROW_NO.	INPUT	PROTEIN_ID	POSITION	RESIDUE_REF	RESIDUE_ALT	SCORE	PREDICTION (cutoff=-2.5)	#SEQ	#CLUSTER	SCORE	PREDICTION (cutoff=0.05)	MEDIAN_INFO	#SEQ
1	ENSP00000321345,124,P,L	ENSP00000321345	124	P	L	-6.91	Deleterious	146	30	29	Damaging	2.79	116
2	ENSP00000321345,126,D,N	ENSP00000321345	126	D	N	-2.84	Deleterious	146	30	4	Damaging	2.79	116
3	ENSP00000321345,126,D,H	ENSP00000321345	126	D	H	-3.89	Deleterious	146	30	2	Damaging	2.79	116
4	ENSP00000321345,126,D,Y	ENSP00000321345	126	D	Y	-5.48	Deleterious	146	30	1	Damaging	2.79	116
5	ENSP00000321345,126,D,G	ENSP00000321345	126	D	G	-3.99	Deleterious	146	30	4	Damaging	2.79	116
6	ENSP00000321345,129,D,E	ENSP00000321345	129	D	E	1.12	Neutral	146	30	929	Tolerated	2.79	116
7	ENSP00000321345,131,V,A	ENSP00000321345	131	V	A	-2.64	Deleterious	146	30	4	Damaging	2.79	116
8	ENSP00000321345,132,T,N	ENSP00000321345	132	T	N	-2.09	Neutral	146	30	26	Damaging	2.79	116
9	ENSP00000321345,132,T,I	ENSP00000321345	132	T	I	-3.94	Deleterious	146	30	2	Damaging	2.79	116
10	ENSP00000321345,135,I,F	ENSP00000321345	135	I	F	-1.93	Neutral	146	30	16	Damaging	2.79	117
11	ENSP00000321345,135,I,T	ENSP00000321345	135	I	T	-1.39	Neutral	146	30	51	Tolerated	2.79	117

```
AACATGACTTGCACCTGGAATGCTGGGAAGCTCACCTACATAGACACAAAATACGTGGTA 480
 N   M   T   C   T   W   N   A   G   K   L   T   Y   I   D   T   K   Y   V   V  160
        TCT     ATC         GGTAGGACGTTC                        GAGGCA
        (S)     (I)         (G) (R) (T) (F)                     (E) (A)
        89      90          91  93  94  96                      97 100
                            GTT     AGG                         GCG
                            (V)     (R)                         (A)
                            92      95                          98
                                                                ATG
                                                                (M)
                                                                99
```

89 - rs1454299797 – no publications, not in ClinVar - T143S
90 - rs1182846342 – no publications, not in ClinVar – T145I
91 - rs759049474 – no publications, not in ClinVar - A148G
92 - rs759049474 – no publications, not in ClinVar – A148V
93 - rs76418789 – 8 publications, not in ClinVar - G149R
94 - rs775258971 – no publications, not in ClinVar – K150T
95 - rs775258971 – no publications, not in ClinVar - K150R
96 - rs776235812 – no publications, not in ClinVar – L151F
97 - rs149318636 – no publications, not in ClinVar – V159E
98 - rs149318636 – no publications, not in ClinVar – V159A
99 - rs555691349 – no publications, not in ClinVar – V159M
100 - rs201902670 – no publications, not in ClinVar – V160A

#ROW_NO.	INPUT	PROTEIN_ID	POSITION	RESIDUE_REF	RESIDUE_ALT	SCORE	PREDICTION (cutoff=-2.5)	#SEQ	#CLUSTER	SCORE	PREDICTION (cutoff=0.05)	MEDIAN_INFO	#SEQ
1	ENSP00000321345,143,T,S	ENSP00000321345	143	T	S	-1.40	Neutral	146	30	138	Tolerated	2.79	117
2	ENSP00000321345,145,T,I	ENSP00000321345	145	T	I	-3.79	Deleterious	146	30	21	Damaging	2.79	117
3	ENSP00000321345,148,A,G	ENSP00000321345	148	A	G	-1.75	Neutral	146	30	254	Tolerated	2.80	122
4	ENSP00000321345,148,A,V	ENSP00000321345	148	A	V	-1.84	Neutral	146	30	140	Tolerated	2.80	122
5	ENSP00000321345,149,G,R	ENSP00000321345	149	G	R	-7.86	Deleterious	146	30	0	Damaging	2.80	122
6	ENSP00000321345,150,K,T	ENSP00000321345	150	K	T	-3.13	Deleterious	146	30	9	Damaging	2.80	122
7	ENSP00000321345,150,K,R	ENSP00000321345	150	K	R	0.42	Neutral	146	30	799	Tolerated	2.80	122
8	ENSP00000321345,151,L,F	ENSP00000321345	151	L	F	-0.38	Neutral	146	30	393	Tolerated	2.80	122
9	ENSP00000321345,159,V,E	ENSP00000321345	159	V	E	-1.98	Neutral	146	30	66	Tolerated	2.79	115
10	ENSP00000321345,159,V,A	ENSP00000321345	159	V	A	-0.93	Neutral	146	30	48	Damaging	2.79	115
11	ENSP00000321345,159,V,M	ENSP00000321345	159	V	M	-1.18	Neutral	146	30	7	Damaging	2.79	115
12	ENSP00000321345,160,V,A	ENSP00000321345	160	V	A	-2.52	Deleterious	146	30	3	Damaging	2.83	109

CATGTGAAGAGTTTAGAGACAGAAGAAGAGCAACAGTATCTCACCTCAAGCTATATTAAC 540
H V K S L E T E E E Q Q Y L T S S Y I N 180

CGTTTG (R) (L) 101 102 | AATTTC (N) (F) 103 104 | GCAGGAAAA (A) (G) (K) 105 107 108 | TCA (S) 106 | CATGCTAAC (H) (V) (N) 109 110 111 | GGC (G) 112 | ACTAGC (T) (S) 113 115 | GTT (V) 114

101 - rs376377228 – no publications, not in ClinVar - H161R
102 - rs1400575409 – no publications, not in ClinVar – V162L
103 - rs777727118 – no publications, not in ClinVar – S164N
104 - rs1282657856 – no publications, not in ClinVar – L165F
105 - rs1256833458 – no publications, not in ClinVar - T167A
106 - rs1256833458 – no publications, not in ClinVar – T167S
107 - rs745884986 – no publications, not in ClinVar – E168G
108 - rs769559634 – no publications, not in ClinVar – E169K
109 - rs371531867 – 1 publication, not in ClinVar - Y173H
110 - rs768316676 – no publications, not in ClinVar – L174V
111 - rs11465797 – 1 publication, not in ClinVar - T175N
112 - rs774037829 – no publications, not in ClinVar - S177G
113 - rs562842287 – no publications, not in ClinVar – I179T
114 - rs772488535 – no publications, not in ClinVar – I179V
115 - rs1036802029 – no publications, not in ClinVar – N180S

#ROW_NO.	INPUT	PROTEIN_ID	POSITION	RESIDUE_REF	RESIDUE_ALT	SCORE	PREDICTION (cutoff=-2.5)	#SEQ	#CLUSTER	SCORE	PREDICTION (cutoff=0.05)	MEDIAN_INFO	#SEQ
1	ENSP00000321345,161,H,R	ENSP00000321345	161	H	R	-1.96	Neutral	146	30	144	Tolerated	2.82	110
2	ENSP00000321345,162,V,L	ENSP00000321345	162	V	L	-0.55	Neutral	146	30	388	Tolerated	2.81	114
3	ENSP00000321345,164,S,N	ENSP00000321345	164	S	N	-0.65	Neutral	146	30	121	Tolerated	2.80	122
4	ENSP00000321345,165,L,F	ENSP00000321345	165	L	F	-2.15	Neutral	146	30	63	Tolerated	2.80	121
5	ENSP00000321345,167,T,A	ENSP00000321345	167	T	A	-2.03	Neutral	146	30	87	Tolerated	2.80	121
6	ENSP00000321345,167,T,S	ENSP00000321345	167	T	S	-1.34	Neutral	146	30	49	Damaging	2.80	121
7	ENSP00000321345,168,E,G	ENSP00000321345	168	E	G	-0.60	Neutral	146	30	371	Tolerated	2.80	121
8	ENSP00000321345,169,E,K	ENSP00000321345	169	E	K	-1.48	Neutral	146	30	48	Damaging	2.80	121
9	ENSP00000321345,173,Y,H	ENSP00000321345	173	Y	H	-1.56	Neutral	146	30	56	Tolerated	2.80	121
10	ENSP00000321345,175,T,N	ENSP00000321345	175	T	N	-1.51	Neutral	146	30	237	Tolerated	2.80	121
11	ENSP00000321345,177,S,G	ENSP00000321345	177	S	G	-0.15	Neutral	146	30	401	Tolerated	2.80	121
12	ENSP00000321345,179,I,T	ENSP00000321345	179	I	T	-2.39	Neutral	146	30	29	Damaging	2.80	121
13	ENSP00000321345,179,I,V	ENSP00000321345	179	I	V	-0.24	Neutral	146	30	752	Tolerated	2.80	121
14	ENSP00000321345,180,N,S	ENSP00000321345	180	N	S	-1.77	Neutral	146	30	127	Tolerated	2.80	121

ATCTCCACTGATTCATTACAAGGTGGCAAGAAGTACTTGGTTTGGGTCCAAGCAGCAAAC 600

I S T D S L Q G G K K Y L V W V Q A A N 200

ACC (T) 116 | AATTTA (N) (L) 117 118 | GAT (D) 119 | CAG (Q) 120 | TTT (F) 121 | TTG (L) 123 | GTA (V) 125

TCG (S) 122 | TGC (C) 124

116 - rs1465880569 – no publications, not in ClinVar - S182T
117 - rs761263134 – no publications, not in ClinVar – D184N
118 - rs868402753 – no publications, not in ClinVar – S185L
119 - rs1376376318 – no publications, not in ClinVar - G188D
120 - rs1383129366 – no publications, not in ClinVar – K191Q
121 - rs146440064 – no publications, not in ClinVar – L193F
122 - rs1558240308 – no publications, not in ClinVar – L193S
123 - rs759642208 – no publications, not in ClinVar - W195L
124 - rs995219905 – no publications, not in ClinVar – W195C
125 - rs143130647 – no publications, not in ClinVar - A199V

#ROW_NO.	INPUT	PROTEIN_ID	POSITION	RESIDUE_REF	RESIDUE_ALT	SCORE	PREDICTION (cutoff=-2.5)	#SEQ	#CLUSTER	SCORE	PREDICTION (cutoff=0.05)	MEDIAN_INFO	#SEQ
1	ENSP00000321345,182,S,T	ENSP00000321345	182	S	T	-1.01	Neutral	146	30	213	Tolerated	2.80	121
2	ENSP00000321345,184,D,N	ENSP00000321345	184	D	N	-1.04	Neutral	146	30	340	Tolerated	2.80	114
3	ENSP00000321345,185,S,L	ENSP00000321345	185	S	L	-1.37	Neutral	146	30	168	Tolerated	2.85	110
4	ENSP00000321345,188,G,D	ENSP00000321345	188	G	D	-1.36	Neutral	146	30	47	Damaging	2.84	98
5	ENSP00000321345,191,K,Q	ENSP00000321345	191	K	Q	-0.90	Neutral	146	30	241	Tolerated	2.81	80
6	ENSP00000321345,193,L,F	ENSP00000321345	193	L	F	-0.46	Neutral	146	30	615	Tolerated	2.81	88
7	ENSP00000321345,193,L,S	ENSP00000321345	193	L	S	0.15	Neutral	146	30	396	Tolerated	2.81	88
8	ENSP00000321345,195,W,L	ENSP00000321345	195	W	L	-7.78	Deleterious	146	30	45	Damaging	2.80	121
9	ENSP00000321345,195,W,C	ENSP00000321345	195	W	C	-8.03	Deleterious	146	30	12	Damaging	2.80	121
10	ENSP00000321345,199,A,V	ENSP00000321345	199	A	V	-1.01	Neutral	146	30	432	Tolerated	2.80	121

```
GCACTAGGCATGGAAGAGTCAAAACAACTGCAAATTCACCTGGATGATATAGTGATACCT 660
 A  L  G  M  E  E  S  K  Q  L  Q  I  H  L  D  D  I  V  I  P  220
ACACCAAGCACGGTAAAG                                AATGTA   TTA---
(T) (P) (S) (T) (V) (K)                            (N) (V)  (L)
126 127 128 129 130 131                            132 133  135 137
                                                      ACA
                                                      (T)
                                                      134

                                                   ATAGTTTTGATA
                                                    I  V  L  I
                                                         TTT
                                                    I219-P220L
                                                         136
```

126 - rs758690026 – no publications, not in ClinVar – A201T
127 - rs1294334604 – no publications, not in ClinVar – L202P
128 - rs961645682 – no publications, not in ClinVar – G203S
129 - rs767358325 – no publications, not in ClinVar – M204T
130 - rs376725743 – no publications, not in ClinVar – E205V
131 - rs560597647 – no publications, not in ClinVar – E206K
132 - rs779805449 – no publications, not in ClinVar – D216N
133 - rs749224934 – no publications, not in ClinVar – I217V
134 - rs1448437790 – no publications, not in ClinVar – I217T
135 - rs1186531124 – no publications, not in ClinVar – I219L
136 - rs1252101276 – no publication, not in Clinvar – I219-P220L (TTT) Insertion
137 - rs1364317777 – no publications, not in ClinVar - P220del - in-frame deletion

#ROW_NO.	INPUT	PROTEIN_ID	POSITION	RESIDUE_REF	RESIDUE_ALT	SCORE	PREDICTION (cutoff=-2.5)	#SEQ	#CLUSTER	SCORE	PREDICTION (cutoff=0.05)	MEDIAN_INFO	#SEQ
1	ENSP00000321345,201,A,T	ENSP00000321345	201	A	T	-1.99	Neutral	146	30	24	Damaging	2.80	121
2	ENSP00000321345,202,L,P	ENSP00000321345	202	L	P	-6.78	Deleterious	146	30	0	Damaging	2.80	121
3	ENSP00000321345,203,G,S	ENSP00000321345	203	G	S	-5.90	Deleterious	146	30	0	Damaging	2.80	121
4	ENSP00000321345,204,M,T	ENSP00000321345	204	M	T	-0.16	Neutral	146	30	1.000	Tolerated	2.80	121
5	ENSP00000321345,205,E,V	ENSP00000321345	205	E	V	-1.91	Neutral	146	30	483	Tolerated	2.80	121
6	ENSP00000321345,206,E,K	ENSP00000321345	206	E	K	-1.48	Neutral	146	30	539	Tolerated	2.80	121
7	ENSP00000321345,216,D,N	ENSP00000321345	216	D	N	-3.41	Deleterious	146	30	42	Damaging	2.80	121
8	ENSP00000321345,217,I,V	ENSP00000321345	217	I	V	-0.55	Neutral	146	30	89	Tolerated	2.80	121
9	ENSP00000321345,217,I,T	ENSP00000321345	217	I	T	-3.89	Deleterious	146	30	2	Damaging	2.80	121
10	ENSP00000321345,219,I,L	ENSP00000321345	219	I	L	-0.44	Neutral	146	30	131	Tolerated	2.79	120

TCTGCAGCCGTC**ATT**TCC**AGG**GCTGAG**ACTATA**AATGCT**ACAGTGCCCAAG**ACC**ATA**ATT 720

S A A V I S R A E T I N A T V P K T I I 240

TTTGTA (F) (V) 138 139
TCA (S) 140
ATG (M) 141
GGG (G) 142
AATGTA (N) (V) 143 144
GCAGAGACCGAG (A) (E) (T) (E) 145 146 147 148
GTA (V) 149
ACA (T) 150
ATG (M) 151

138 - rs575018195 – no publications, not in ClinVar - S221F
139 - rs1558243571 – no publications, not in ClinVar – A222V
140 - rs1570848374 – no publications, not in ClinVar – A222S
141 - rs753835420 – no publications, not in ClinVar – I225M
142 - rs1308075262 – no publications, not in ClinVar – R227G
143 - rs1237612829 – no publications, not in ClinVar – T230N
144 - rs778411499 – no publications, not in ClinVar – I231V
145 - rs758102857 – no publications, not in ClinVar – T234A
146 - rs1264652668 – no publications, not in ClinVar – V235E
147 - rs868018244 – no publications, not in ClinVar – P236T
148 - rs1358774745 – no publications, not in ClinVar – K237E
149 - rs777244526 – no publications, not in ClinVar – I239V
150 - rs1160630933 – no publications, not in ClinVar – I239T
151 - rs1558243635 – no publications, not in ClinVar – I239M

#ROW_NO.	INPUT	PROTEIN_ID	POSITION	RESIDUE_REF	RESIDUE_ALT	SCORE	PREDICTION (cutoff=-2.5)	#SEQ	#CLUSTER	SCORE	PREDICTION (cutoff=0.05)	MEDIAN_INFO	#SEQ
1	ENSP00000321345,221,S,F	ENSP00000321345	221	S	F	-1.77	Neutral	146	30	107	Tolerated	2.79	120
2	ENSP00000321345,222,A,V	ENSP00000321345	222	A	V	-1.27	Neutral	146	30	356	Tolerated	2.79	120
3	ENSP00000321345,222,A,S	ENSP00000321345	222	A	S	-0.82	Neutral	146	30	169	Tolerated	2.79	120
4	ENSP00000321345,225,I,M	ENSP00000321345	225	I	M	-1.44	Neutral	146	30	14	Damaging	2.80	119
5	ENSP00000321345,227,R,G	ENSP00000321345	227	R	G	-2.64	Deleterious	146	30	103	Tolerated	2.79	120
6	ENSP00000321345,230,T,N	ENSP00000321345	230	T	N	-0.49	Neutral	146	30	677	Tolerated	2.79	120
7	ENSP00000321345,231,I,V	ENSP00000321345	231	I	V	-0.11	Neutral	146	30	512	Tolerated	2.79	111
8	ENSP00000321345,234,T,A	ENSP00000321345	234	T	A	-1.06	Neutral	146	30	25	Damaging	2.79	120
9	ENSP00000321345,235,V,E	ENSP00000321345	235	V	E	-1.95	Neutral	146	30	38	Damaging	2.81	118
10	ENSP00000321345,236,P,T	ENSP00000321345	236	P	T	-2.43	Neutral	146	30	24	Damaging	2.81	117
11	ENSP00000321345,237,K,E	ENSP00000321345	237	K	E	-0.94	Neutral	146	30	66	Tolerated	2.81	108
12	ENSP00000321345,239,I,V	ENSP00000321345	239	I	V	0.07	Neutral	146	30	323	Tolerated	2.85	107
13	ENSP00000321345,239,I,T	ENSP00000321345	239	I	T	-0.14	Neutral	146	30	333	Tolerated	2.85	107
14	ENSP00000321345,239,I,M	ENSP00000321345	239	I	M	-0.85	Neutral	146	30	9	Damaging	2.85	107

```
TATTGGGATAGTCAAACAACAATTGAAAAGGTTTCCTGTGAAATGAGATACAAGGCTACA 780
 Y  W  D  S  Q  T  T  I  E  K  V  S  C  E  M  R  Y  K  A  T  260
    TGT         AAAAAAATAAGT   AATGAT         GGA         CACGAGGTCATA
    (C)         (K) (K) (I) (S) (N) (D)       (G)         (H) (E) (V) (I)
    152         153 154 156 157 158 159       161         162 163 165 166
                    AGA            GCT                        AAC
                    (R)            (A)                        (N)
                    155            160                        164
```

152 - rs369986474 – no publications, not in ClinVar – W242C
153 - rs1411124273 – no publications, not in ClinVar - Q245K
154 - rs1023709758 – no publications, not in ClinVar – T246K
155 - rs1023709758 – no publications, not in ClinVar – T246R
156 - rs770357159 – no publications, not in ClinVar – T247I
157 - rs1326898192 – no publications, not in ClinVar – I248S
158 - rs970390731 – no publications, not in ClinVar - K250N
159 - rs368389094 – no publications, not in ClinVar – V251D
160 - rs368389094 – no publications, not in ClinVar – V251A
161 - rs1444664057 – no publications, not in ClinVar – E254G
162 - rs1490421226 – no publications, not in ClinVar – Y257H
163 - rs763076695 – no publications, not in ClinVar – K258E
164 - rs1308394223 – no publications, not in ClinVar – K258N
165 - rs1298953490 – no publications, not in ClinVar – A259V
166 - rs180809196 – no publications, not in ClinVar – T260I

#ROW_NO.	INPUT	PROTEIN_ID	POSITION	RESIDUE_REF	RESIDUE_ALT	SCORE	PREDICTION (cutoff=-2.5)	#SEQ	#CLUSTER	SCORE	PREDICTION (cutoff=0.05)	MEDIAN_INFO	#SEQ
1	ENSP00000321345,242,W,C	ENSP00000321345	242	W	C	-9.31	Deleterious	146	30	1	Damaging	2.79	118
2	ENSP00000321345,245,Q,K	ENSP00000321345	245	Q	K	-0.76	Neutral	146	30	507	Tolerated	2.79	119
3	ENSP00000321345,246,T,K	ENSP00000321345	246	T	K	-2.19	Neutral	146	30	226	Tolerated	2.79	119
4	ENSP00000321345,246,T,R	ENSP00000321345	246	T	R	-2.29	Neutral	146	30	85	Tolerated	2.79	119
5	ENSP00000321345,247,T,I	ENSP00000321345	247	T	I	-0.77	Neutral	146	30	396	Tolerated	2.79	119
6	ENSP00000321345,248,I,S	ENSP00000321345	248	I	S	-1.95	Neutral	146	30	72	Tolerated	2.80	118
7	ENSP00000321345,250,K,N	ENSP00000321345	250	K	N	-0.16	Neutral	146	30	452	Tolerated	2.84	86
8	ENSP00000321345,251,V,D	ENSP00000321345	251	V	D	-3.15	Deleterious	146	30	1	Damaging	2.81	116
9	ENSP00000321345,251,V,A	ENSP00000321345	251	V	A	-1.82	Neutral	146	30	49	Damaging	2.81	116
10	ENSP00000321345,254,E,G	ENSP00000321345	254	E	G	-3.68	Deleterious	146	30	14	Damaging	2.79	119
11	ENSP00000321345,257,Y,H	ENSP00000321345	257	Y	H	-3.16	Deleterious	146	30	138	Tolerated	2.80	120
12	ENSP00000321345,258,K,E	ENSP00000321345	258	K	E	-2.25	Neutral	146	30	5	Damaging	2.80	120
13	ENSP00000321345,258,K,N	ENSP00000321345	258	K	N	-2.75	Deleterious	146	30	3	Damaging	2.80	120
14	ENSP00000321345,259,A,V	ENSP00000321345	259	A	V	-1.89	Neutral	146	30	259	Tolerated	2.81	119
15	ENSP00000321345,260,T,I	ENSP00000321345	260	T	I	-2.31	Neutral	146	30	41	Damaging	2.80	120

```
ACACACCAAACTTGGAATGTTAAAGAATTTGACACCAATTTTACATATGTGCAACAGTCA 840
 T  N  Q  T  W  N  V  K  E  F  D  T  N  F  T  Y  V  Q  Q  S  280
AAAACCAAA          ATT    CAA    GGCAAC    TGTATATGTATG    GAG
(K)(H)(K)          (I)    (Q)    (G)(N)    (C)(I)(C)(M)    (E)
167 168 169        170    172    173 174   175 176 177 178 180
                   TTT                              GCG
                   (F)                              (A)
                   171                              179
```

```
167 - rs774474216     - no publications, not in ClinVar - T261K
168 - rs1274317659    - no publications, not in ClinVar - N262H
169 - rs1558243703    - no publications, not in ClinVar - Q263K
170 - rs542279299     - no publications, not in ClinVar - V267I
171 - rs542279299     - no publications, not in ClinVar - V267F
172 - rs773477018     - no publications, not in ClinVar - E269Q
173 - rs1247971721    - no publications, not in ClinVar - D271G
174 - rs1308466742    - no publications, not in ClinVar - T272N
175 - rs980481356     - no publications, not in ClinVar - F274C
176 - rs1250252334    - no publications, not in ClinVar - T275I
177 - rs941400363     - no publications, not in ClinVar - Y276C
178 - rs762813338     - no publications, not in ClinVar - V277M
179 - rs1405024139    - no publications, not in ClinVar - V277A
180 - rs1244428651    - no publications, not in ClinVar - Q279E
```

#ROW_NO.	INPUT	PROTEIN_ID	POSITION	RESIDUE_REF	RESIDUE_ALT	SCORE	PREDICTION (cutoff=-2.5)	#SEQ	#CLUSTER	SCORE	PREDICTION (cutoff=0.05)	MEDIAN_INFO	#SEQ
1	ENSP00000321345,261,T,K	ENSP00000321345	261	T	K	-1.06	Neutral	146	30	182	Tolerated	2.80	120
2	ENSP00000321345,262,N,H	ENSP00000321345	262	N	H	-2.39	Neutral	146	30	24	Damaging	2.80	120
3	ENSP00000321345,263,Q,K	ENSP00000321345	263	Q	K	-0.90	Neutral	146	30	243	Tolerated	2.80	120
4	ENSP00000321345,267,V,I	ENSP00000321345	267	V	I	-0.40	Neutral	146	30	160	Tolerated	2.82	113
5	ENSP00000321345,267,V,F	ENSP00000321345	267	V	F	-2.59	Deleterious	146	30	2	Damaging	2.82	113
6	ENSP00000321345,269,E,Q	ENSP00000321345	269	E	Q	-1.09	Neutral	146	30	72	Tolerated	2.84	95
7	ENSP00000321345,271,D,G	ENSP00000321345	271	D	G	-1.85	Neutral	146	30	16	Damaging	2.84	106
8	ENSP00000321345,272,T,N	ENSP00000321345	272	T	N	-1.53	Neutral	146	30	3	Damaging	2.80	117
9	ENSP00000321345,274,F,C	ENSP00000321345	274	F	C	-1.07	Neutral	146	30	107	Tolerated	2.79	119
10	ENSP00000321345,275,T,I	ENSP00000321345	275	T	I	-2.70	Deleterious	146	30	12	Damaging	2.79	119
11	ENSP00000321345,276,Y,C	ENSP00000321345	276	Y	C	-1.89	Neutral	146	30	69	Tolerated	2.79	115
12	ENSP00000321345,277,V,M	ENSP00000321345	277	V	M	-0.58	Neutral	146	30	126	Tolerated	2.79	119
13	ENSP00000321345,277,V,A	ENSP00000321345	277	V	A	0.29	Neutral	146	30	800	Tolerated	2.79	119
14	ENSP00000321345,279,Q,E	ENSP00000321345	279	Q	E	-0.82	Neutral	146	30	274	Tolerated	2.79	119

GAATTCTAC**TTGGAG**CCA**AACATT**AAGTAC**GTA**TTTCAA**GTGAGA**TGTCAAGAAACA**GGC** 900
E F Y L E P N I K Y V F Q V R C Q E T G 300

TGGAAG (W) (K) 181 182
AAGTTT (K) (F) 183 185
AAT (S) 184
ATA (I) 186
CTAAGT (L) (S) 187 189
GGG (G) 188
CGC (R) 190
GAC (D) 191
GTC (V) 192

181 - rs1312712224 – no publications, not in ClinVar - L284W
182 - rs1423971926 – no publications, not in ClinVar – E285K
183 - rs773754315 – no publications, not in ClinVar – N287K
184 - rs773754315 – no publications, not in ClinVar – N287S
185 - rs1400909129 – no publications, not in ClinVar – I288F
186 - rs767104591 – no publications, not in ClinVar - V291I
187 - rs1338228352 – no publications, not in ClinVar - V294L
188 - rs1392327910 – no publications, not in ClinVar - V294G
189 - rs965693876 – no publications, not in ClinVar - R295S
190 - rs750164194 – no publications, not in ClinVar - G300R
191 - rs755811354 – no publications, not in ClinVar – G300D
192 - rs755811354 – no publications, not in ClinVar – G300V

#ROW_NO.	INPUT	PROTEIN_ID	POSITION	RESIDUE_REF	RESIDUE_ALT	SCORE	PREDICTION (cutoff=-2.5)	#SEQ	#CLUSTER	SCORE	PREDICTION (cutoff=0.05)	MEDIAN_INFO	#SEQ
1	ENSP00000321345,284,L,W	ENSP00000321345	284	L	W	-5.07	Deleterious	146	30	0	Damaging	2.79	118
2	ENSP00000321345,285,E,K	ENSP00000321345	285	E	K	-0.38	Neutral	146	30	452	Tolerated	2.79	118
3	ENSP00000321345,287,N,K	ENSP00000321345	287	N	K	-1.17	Neutral	146	30	308	Tolerated	2.79	118
4	ENSP00000321345,287,N,S	ENSP00000321345	287	N	S	-0.57	Neutral	146	30	401	Tolerated	2.79	118
5	ENSP00000321345,288,I,F	ENSP00000321345	288	I	F	-1.23	Neutral	146	30	4	Damaging	2.79	119
6	ENSP00000321345,291,V,I	ENSP00000321345	291	V	I	-0.73	Neutral	146	30	434	Tolerated	2.79	119
7	ENSP00000321345,294,V,L	ENSP00000321345	294	V	L	-1.14	Neutral	146	30	21	Damaging	2.79	119
8	ENSP00000321345,295,R,S	ENSP00000321345	295	R	S	-2.55	Deleterious	146	30	239	Tolerated	2.81	118
9	ENSP00000321345,300,G,R	ENSP00000321345	300	G	R	-4.14	Deleterious	146	30	6	Damaging	2.81	118
10	ENSP00000321345,300,G,D	ENSP00000321345	300	G	D	-3.19	Deleterious	146	30	5	Damaging	2.81	118
11	ENSP00000321345,300,G,V	ENSP00000321345	300	G	V	-4.56	Deleterious	146	30	8	Damaging	2.81	118

```
AAAAGGTACTGGCAGCCTTGGAGTTCACTGTTTTTTCATAAAACACCTGAAACAGTTCCC 960
 K  R  Y  W  Q  P  W  S  S  L  F  F  H  K  T  P  E  T  V  P  320
CAA               GCTGGGCGT   CCG    CTTCGT    GCACATGCAATAATT
(Q)               (A)(G)(R)   (P)    (L)(R)    (A)(H)(A)(I)(I)
193               194 196 197 198    201 202   204 205 206 208 209
                  TCT         CAG       TAT           GTA    CTT
                  (S)         (Q)       (Y)           (V)    (L)
                  195         199       203           207    210
                              ATG
                              (M)
                              200
```

193 - rs1450509119 – no publications, not in ClinVar - K301Q
194 - rs545141199 – no publications, not in ClinVar - P306A
195 - rs545141199 – no publications, not in ClinVar – P306S
196 - rs779430236 – no publications, not in ClinVar - W307G
197 - rs748585783 – no publications, not in ClinVar - S308R
198 - rs7530511 – 39 publications, not in ClinVar - L310P
199 - rs7530511 – 39 publications, not in ClinVar - L310Q
200 - rs777827669 – no publications, not in ClinVar - L310M
201 - rs1570874417 – no publications, not in ClinVar - F312L
202 - rs749061229 – no publications, not in ClinVar – H313R
203 - rs1268199686 – no publications, not in ClinVar – H313Y
204 - rs768312080 – no publications, not in ClinVar - T315A
205 - rs1190102539 – no publications, not in ClinVar - P316H
206 - rs767190530 – no publications, not in ClinVar - E317A
207 - rs767190530 – no publications, not in ClinVar - E317V
208 - rs772786061 – no publications, not in ClinVar - T318I
209 - rs760405559 – no publications, not in ClinVar - V319I
210 - rs760405559 – no publications, not in ClinVar – V319L

#ROW_NO.	INPUT	PROTEIN_ID	POSITION	RESIDUE_REF	RESIDUE_ALT	SCORE	PREDICTION (cutoff=-2.5)	#SEQ	#CLUSTER	SCORE	PREDICTION (cutoff=0.05)	MEDIAN_INFO	#SEQ
1	ENSP00000321345,301,K,Q	ENSP00000321345	301	K	Q	-1.16	Neutral	146	30	70	Tolerated	2.81	118
2	ENSP00000321345,306,P,A	ENSP00000321345	306	P	A	-1.46	Neutral	146	30	440	Tolerated	2.83	117
3	ENSP00000321345,306,P,S	ENSP00000321345	306	P	S	-0.14	Neutral	146	30	417	Tolerated	2.83	117
4	ENSP00000321345,307,W,G	ENSP00000321345	307	W	G	-10.85	Deleterious	146	30	0	Damaging	2.83	117
5	ENSP00000321345,308,S,R	ENSP00000321345	308	S	R	-4.26	Deleterious	146	30	1	Damaging	2.83	117
6	ENSP00000321345,310,L,P	ENSP00000321345	310	L	P	3.73	Neutral	146	30	1.000	Tolerated	2.90	70
7	ENSP00000321345,310,L,Q	ENSP00000321345	310	L	Q	1.36	Neutral	146	30	2	Damaging	2.90	70
8	ENSP00000321345,310,L,M	ENSP00000321345	310	L	M	0.53	Neutral	146	30	2	Damaging	2.90	70
9	ENSP00000321345,312,F,L	ENSP00000321345	312	F	L	-1.08	Neutral	146	30	153	Tolerated	2.89	72
10	ENSP00000321345,313,H,R	ENSP00000321345	313	H	R	-1.94	Neutral	146	30	165	Tolerated	2.89	72
11	ENSP00000321345,313,H,Y	ENSP00000321345	313	H	Y	-1.17	Neutral	146	30	289	Tolerated	2.89	72
12	ENSP00000321345,315,T,A	ENSP00000321345	315	T	A	-3.18	Deleterious	146	30	3	Damaging	2.89	72
13	ENSP00000321345,316,P,H	ENSP00000321345	316	P	H	-4.25	Deleterious	146	30	0	Damaging	2.87	73
14	ENSP00000321345,317,E,A	ENSP00000321345	317	E	A	-2.68	Deleterious	146	30	244	Tolerated	2.87	74
15	ENSP00000321345,317,E,V	ENSP00000321345	317	E	V	-3.57	Deleterious	146	30	10	Damaging	2.87	74
16	ENSP00000321345,318,T,I	ENSP00000321345	318	T	I	-2.01	Neutral	146	30	94	Tolerated	2.87	74
17	ENSP00000321345,319,V,I	ENSP00000321345	319	V	I	-0.51	Neutral	146	30	179	Tolerated	2.87	71
18	ENSP00000321345,319,V,L	ENSP00000321345	319	V	L	-0.70	Neutral	146	30	141	Tolerated	2.87	71

```
CAGGTCACATCAAAAGCATTCCAACCATGACACATGGAATTCTGGGCTAACAGTTGCTTCC 1020
 Q  V  T  S  K  A  F  Q  H  D  T  W  N  S  G  L  T  V  A  S  340
               ACATAC   AATGAA              GTG      ATT
               (T)(L)   (N)(E)              (V)      (I)
               211 212  213 214             215      217
                                            AGG      GAT
                                            (R)      (D)
                                            216      218
```

211 - rs776389611 – no publications, not in ClinVar - A326T
212 - rs1162583283 – no publications, not in ClinVar – F327L
213 - rs759109559 – no publications, not in ClinVar - H329N
214 - rs752947237 – no publications, not in ClinVar - D330E
215 - rs752038555 – no publications, not in ClinVar - G335V
216 - rs764546073 – no publications, not in ClinVar - G335R
217 - rs1174013187 – no publications, not in ClinVar - V338I
218 - rs1244790854 – no publications, not in ClinVar - V338D

#ROW_NO.	INPUT	PROTEIN_ID	POSITION	RESIDUE_REF	RESIDUE_ALT	SCORE	PREDICTION (cutoff=-2.5)	#SEQ	#CLUSTER	SCORE	PREDICTION (cutoff=0.05)	MEDIAN_INFO	#SEQ
1	ENSP00000321345,326,A,T	ENSP00000321345	326	A	T	-0.10	Neutral	146	30	196	Tolerated	3.08	32
2	ENSP00000321345,327,F,L	ENSP00000321345	327	F	L	0.48	Neutral	146	30	306	Tolerated	3.14	28
3	ENSP00000321345,329,H,N	ENSP00000321345	329	H	N	-1.49	Neutral	146	30	13	Damaging	3.04	33
4	ENSP00000321345,330,D,E	ENSP00000321345	330	D	E	-1.02	Neutral	146	30	227	Tolerated	3.08	31
5	ENSP00000321345,335,G,V	ENSP00000321345	335	G	V	-1.34	Neutral	146	30	9	Damaging	3.34	29
6	ENSP00000321345,335,G,R	ENSP00000321345	335	G	R	-0.24	Neutral	146	30	15	Damaging	3.34	29
7	ENSP00000321345,338,V,I	ENSP00000321345	338	V	I	-0.35	Neutral	146	30	887	Tolerated	3.34	29
8	ENSP00000321345,338,V,D	ENSP00000321345	338	V	D	-0.45	Neutral	146	30	6	Damaging	3.34	29

ATCTCTACAGGGCACCTTACTTCTGACAACAGAGGAGACATTGGACTTTTATTGGGAATG 1080

I S T G H L T S D N R G D I G L L L G M 360

GTC (V) 219	GCA (A) 221	TTTAAT (F) (N) 222 223	ACT (T) 224	TTT (F) 226	TCG (S) 227	GTG (V) 228
ATG (M) 220			AGT (S) 225			ATT (I) 229

219 - rs373821085 – no publications, not in ClinVar – I341V
220 - rs750715115 – no publications, not in ClinVar – I341M
221 - rs756229051 – no publications, not in ClinVar – T343A
222 - rs1464115526 – no publications, not in ClinVar – L346F
223 - rs1203890607 – no publications, not in ClinVar – T347N
224 - rs754093485 – no publications, not in ClinVar – I354T
225 - rs754093485 – no publications, not in ClinVar – I354S
226 - rs1307957404 – no publications, not in ClinVar – L356F
227 - rs1349400977 – no publications, not in ClinVar – L358S
228 - rs375552098 – no publications, not in ClinVar – M360V
229 - rs751263927 – no publications, not in ClinVar – M360I

#ROW_NO.	INPUT	PROTEIN_ID	POSITION	RESIDUE_REF	RESIDUE_ALT	SCORE	PREDICTION (cutoff=-2.5)	#SEQ	#CLUSTER	SCORE	PREDICTION (cutoff=0.05)	MEDIAN_INFO	#SEQ
1	ENSP00000321345,341,I,V	ENSP00000321345	341	I	V	-0.09	Neutral	146	30	228	Tolerated	3.16	30
2	ENSP00000321345,341,I,M	ENSP00000321345	341	I	M	-0.33	Neutral	146	30	2	Damaging	3.16	30
3	ENSP00000321345,343,T,A	ENSP00000321345	343	T	A	-0.46	Neutral	146	30	164	Tolerated	3.16	30
4	ENSP00000321345,346,L,F	ENSP00000321345	346	L	F	-1.06	Neutral	146	30	156	Tolerated	3.16	30
5	ENSP00000321345,347,T,N	ENSP00000321345	347	T	N	-1.16	Neutral	146	30	52	Tolerated	3.16	30
6	ENSP00000321345,354,I,T	ENSP00000321345	354	I	T	-0.33	Neutral	146	30	227	Tolerated	3.16	30
7	ENSP00000321345,354,I,S	ENSP00000321345	354	I	S	-0.52	Neutral	146	30	20	Damaging	3.16	30
8	ENSP00000321345,356,L,F	ENSP00000321345	356	L	F	-1.46	Neutral	146	30	163	Tolerated	3.33	29
9	ENSP00000321345,358,L,S	ENSP00000321345	358	L	S	0.02	Neutral	146	30	520	Tolerated	3.33	29
10	ENSP00000321345,360,M,V	ENSP00000321345	360	M	V	-1.11	Neutral	146	30	10	Damaging	3.33	29
11	ENSP00000321345,360,M,I	ENSP00000321345	360	M	I	-1.22	Neutral	146	30	8	Damaging	3.33	29

```
ATCGTCTTTGCTGTTATGTTGTCAATTCTTTCTTTGATTGGGATATTTAACAGATCATTC 1140
 I  V  F  A  V  M  L  S  I  L  S  L  I  G  I  F  N  R  S  F  380
ACCATC          ACG          ATTCCTTTCTTT          AAA
(T)(I)          (T)          (I)(P)(F)(F)          (K)
230 231         233          235 236 237 238       240
   TTC          AGG                   ACT
   (F)          (R)                   (T)
   232          234                   239
```

```
230 - rs1302959794    - no publications, not in ClinVar - I361T
231 - rs41313262      - 4 publications, not in ClinVar  - V362I
232 - rs41313262      - no publications, not in ClinVar - V362F
233 - rs1053176114    - no publications, not in ClinVar - M366T
234 - rs1053176114    - no publications, not in ClinVar - M366R
235 - rs1275143809    - no publications, not in ClinVar - L370I
236 - rs757084952     - no publications, not in ClinVar - S371P
237 - rs199542433     - no publications, not in ClinVar - L372F
238 - rs201752419     - no publications, not in ClinVar - I373F
239 - rs775162322     - no publications, not in ClinVar - I373T
240 - rs867213276     - no publications, not in ClinVar - R378K
```

#ROW_NO.	INPUT	PROTEIN_ID	POSITION	RESIDUE_REF	RESIDUE_ALT	SCORE	PREDICTION (cutoff=-2.5)	#SEQ	#CLUSTER	SCORE	PREDICTION (cutoff=0.05)	MEDIAN_INFO	#SEQ
1	ENSP00000321345,361,I,T	ENSP00000321345	361	I	T	-0.27	Neutral	146	30	5	Damaging	3.33	29
2	ENSP00000321345,362,V,I	ENSP00000321345	362	V	I	0.06	Neutral	146	30	303	Tolerated	3.33	29
3	ENSP00000321345,362,V,F	ENSP00000321345	362	V	F	0.24	Neutral	146	30	881	Tolerated	3.33	29
4	ENSP00000321345,366,M,T	ENSP00000321345	366	M	T	0.29	Neutral	146	30	202	Tolerated	3.33	29
5	ENSP00000321345,366,M,R	ENSP00000321345	366	M	R	-0.92	Neutral	146	30	7	Damaging	3.33	29
6	ENSP00000321345,370,L,I	ENSP00000321345	370	L	I	-0.56	Neutral	146	30	69	Tolerated	3.33	29
7	ENSP00000321345,371,S,P	ENSP00000321345	371	S	P	-2.10	Neutral	146	30	37	Damaging	3.33	29
8	ENSP00000321345,372,L,F	ENSP00000321345	372	L	F	-1.82	Neutral	146	30	19	Damaging	3.33	29
9	ENSP00000321345,373,I,F	ENSP00000321345	373	I	F	-1.10	Neutral	146	30	7	Damaging	3.33	29
10	ENSP00000321345,373,I,T	ENSP00000321345	373	I	T	-0.44	Neutral	146	30	175	Tolerated	3.33	29
11	ENSP00000321345,378,R,K	ENSP00000321345	378	R	K	-0.35	Neutral	146	30	29	Damaging	3.33	29

```
CGAACTGGGATTAAAAGAAGGATCTTATTGTTAATACCAAAGTGGCTTTATGAAGATATT 1200
 R  T  G  I  K  R  R  I  L  L  L  I  P  K  W  L  Y  E  D  I  400
CAA     AGG     GAAAAA          TGG             AGGGTT
(Q)     (R)     (E) (K)         (W)             (R) (V)
241     242     243 244         245             246 247
                                                   TTT
                                                   (F)
                                                   248
```

241 - rs11209026 – 210 publications, reported in ClinVar - R381Q
242 - rs1168522773 – no publications, not in ClinVar - G383R
243 - rs547510231 – no publications, not in ClinVar - K385E
244 - rs773635455 – no publications, not in ClinVar - R386K
245 - rs761242932 – no publications, not in ClinVar - L390W
246 - rs776798769 – no publications, not in ClinVar - W395R
247 - rs1463672176 – no publications, not in ClinVar - L396V
248 - rs1463672176 – no publications, not in ClinVar - L396F

#ROW_NO.	INPUT	PROTEIN_ID	POSITION	RESIDUE_REF	RESIDUE_ALT	SCORE	PREDICTION (cutoff=-2.5)	#SEQ	#CLUSTER	SCORE	PREDICTION (cutoff=0.05)	MEDIAN_INFO	#SEQ
1	ENSP00000321345,381,R,Q	ENSP00000321345	381	R	Q	-1.16	Neutral	146	30	0	Damaging	3.33	29
2	ENSP00000321345,383,G,R	ENSP00000321345	383	G	R	-0.83	Neutral	146	30	354	Tolerated	3.33	29
3	ENSP00000321345,385,K,E	ENSP00000321345	385	K	E	-1.06	Neutral	146	30	2	Damaging	3.33	30
4	ENSP00000321345,386,R,K	ENSP00000321345	386	R	K	-0.63	Neutral	146	30	32	Damaging	3.33	30
5	ENSP00000321345,390,L,W	ENSP00000321345	390	L	W	0.00	Neutral	146	30	4	Damaging	3.33	29
6	ENSP00000321345,395,W,R	ENSP00000321345	395	W	R	-4.99	Deleterious	146	30	0	Damaging	3.33	29
7	ENSP00000321345,396,L,V	ENSP00000321345	396	L	V	-0.70	Neutral	146	30	0	Damaging	3.33	29
8	ENSP00000321345,396,L,F	ENSP00000321345	396	L	F	-1.59	Neutral	146	30	0	Damaging	3.33	29

```
CCTAATATGAAAAACAGCAATGTTGTGAAAATGCTACAGGAAAATAGTGAACTTATGAAT 1260
 P  N  M  K  N  S  N  V  V  K  M  L  Q  E  N  S  E  L  M  N  420
TCTGAT                AAAATT AGAATA CACAAA ACT  TTT  AGT
(S)(D)                (K)(I) (R)(I) (H)(K) (T)  (F)  (S)
249 250               251 253 255 256 257 259 260 261 262
                      AACGCT        CGG
                      (N)(A)        (R)
                      252 254       258
```

249 - rs1232743079 – no publications, not in ClinVar - P401S
250 - rs759843168 – no publications, not in ClinVar – N402D
251 - rs1208319440 – no publications, not in ClinVar - N407K
252 - rs1208319440 – no publications, not in ClinVar – N407N
253 - rs1238451416 – no publications, not in ClinVar – V408I
254 - rs1484438152 – no publications, not in ClinVar – V408A
255 - rs267598700 – no publications, not in ClinVar - K410R
256 - rs544186603 – no publications, not in ClinVar – M411I
257 - rs919329759 – no publications, not in ClinVar - Q413H
258 - rs1159199594 – no publications, not in ClinVar - Q413R
259 - rs771501924 – no publications, not in ClinVar - E414K
260 - rs1442474244 – no publications, not in ClinVar - S416T
261 - rs1348768484 – no publications, not in ClinVar – L418F
262 - rs1409219262 – no publications, not in ClinVar - N420S

#ROW_NO.	INPUT	PROTEIN_ID	POSITION	RESIDUE_REF	RESIDUE_ALT	SCORE	PREDICTION (cutoff=-2.5)	#SEQ	#CLUSTER	SCORE	PREDICTION (cutoff=0.05)	MEDIAN_INFO	#SEQ
1	ENSP00000321345,401,P,S	ENSP00000321345	401	P	S	-2.72	Deleterious	146	30	0	Damaging	3.33	29
2	ENSP00000321345,402,N,D	ENSP00000321345	402	N	D	-1.79	Neutral	146	30	54	Tolerated	3.33	29
3	ENSP00000321345,407,N,K	ENSP00000321345	407	N	K	-1.68	Neutral	146	30	130	Tolerated	3.33	29
4	ENSP00000321345,408,V,I	ENSP00000321345	408	V	I	-0.46	Neutral	146	30	174	Tolerated	3.33	29
5	ENSP00000321345,408,V,A	ENSP00000321345	408	V	A	-0.02	Neutral	146	30	648	Tolerated	3.33	29
6	ENSP00000321345,410,K,R	ENSP00000321345	410	K	R	-1.04	Neutral	146	30	7	Damaging	3.33	29
7	ENSP00000321345,411,M,I	ENSP00000321345	411	M	I	-0.19	Neutral	146	30	252	Tolerated	3.33	29
8	ENSP00000321345,413,Q,H	ENSP00000321345	413	Q	H	-1.36	Neutral	146	30	2	Damaging	3.33	29
9	ENSP00000321345,413,Q,R	ENSP00000321345	413	Q	R	-1.31	Neutral	146	30	11	Damaging	3.33	29
10	ENSP00000321345,414,E,K	ENSP00000321345	414	E	K	-1.15	Neutral	146	30	35	Damaging	3.32	30
11	ENSP00000321345,416,S,T	ENSP00000321345	416	S	T	-0.98	Neutral	146	30	16	Damaging	3.32	28
12	ENSP00000321345,418,L,F	ENSP00000321345	418	L	F	1.46	Neutral	146	30	1.000	Tolerated	3.32	28
13	ENSP00000321345,420,N,S	ENSP00000321345	420	N	S	-0.80	Neutral	146	30	41	Damaging	3.32	28

```
AATAATTCCAGTGAGCAGGTCCTATATGTTGATCCCATGATTACAGAGATAAAAGAAATC 1320
 N  N  S  S  E  Q  V  L  Y  V  D  P  M  I  T  E  I  K  E  I  440
ACTGAT          AAGCCGTTCCCA        GAA     ACGGTTAAA            GTC
(T)(D)          (K)(P)(F)(P)        (E)     (T)(V)(K)            (V)
263 264         265 266 267 268     269     270 271 273          274
                                               ACT
                                               (T)
                                               272
```

263 - rs759640556 - no publications, not in ClinVar - N421T
264 - rs1016412497 - no publications, not in ClinVar - N422D
265 - rs769973852 - no publications, not in ClinVar - E425K
266 - rs1558269449 - no publications, not in ClinVar - Q426P
267 - rs371737947 - no publications, not in ClinVar - V427F
268 - rs113281071 - no publications, not in ClinVar - L428P
269 - rs199885679 - no publications, not in ClinVar - D431E
270 - rs1481789783 - no publications, not in ClinVar - M433T
271 - rs958559603 - no publications, not in ClinVar - I434V
272 - rs1246947739 - no publications, not in ClinVar - I434T
273 - rs760637251 - no publications, not in ClinVar - T435K
274 - rs1449828831 - no publications, not in ClinVar - I440V

#ROW_NO.	INPUT	PROTEIN_ID	POSITION	RESIDUE_REF	RESIDUE_ALT	SCORE	PREDICTION (cutoff=-2.5)	#SEQ	#CLUSTER	SCORE	PREDICTION (cutoff=0.05)	MEDIAN_INFO	#SEQ
1	ENSP00000321345,421,N,T	ENSP00000321345	421	N	T	-0.69	Neutral	146	30	52	Tolerated	3.32	28
2	ENSP00000321345,422,N,D	ENSP00000321345	422	N	D	-1.00	Neutral	146	30	227	Tolerated	3.32	28
3	ENSP00000321345,425,E,K	ENSP00000321345	425	E	K	-1.40	Neutral	146	30	11	Damaging	3.32	28
4	ENSP00000321345,426,Q,P	ENSP00000321345	426	Q	P	-1.35	Neutral	146	30	5	Damaging	3.32	28
5	ENSP00000321345,427,V,F	ENSP00000321345	427	V	F	-1.55	Neutral	146	30	230	Tolerated	3.32	28
6	ENSP00000321345,428,L,P	ENSP00000321345	428	L	P	-1.34	Neutral	146	30	78	Tolerated	3.32	28
7	ENSP00000321345,431,D,E	ENSP00000321345	431	D	E	-1.41	Neutral	146	30	0	Damaging	3.32	28
8	ENSP00000321345,433,M,T	ENSP00000321345	433	M	T	-0.16	Neutral	146	30	216	Tolerated	3.32	28
9	ENSP00000321345,434,I,V	ENSP00000321345	434	I	V	-0.21	Neutral	146	30	116	Tolerated	3.32	28
10	ENSP00000321345,434,I,T	ENSP00000321345	434	I	T	-1.55	Neutral	146	30	1	Damaging	3.32	28
11	ENSP00000321345,435,T,K	ENSP00000321345	435	T	K	-2.33	Neutral	146	30	0	Damaging	3.32	28
12	ENSP00000321345,440,I,V	ENSP00000321345	440	I	V	0.08	Neutral	146	30	272	Tolerated	3.32	28

```
TTCATCCCAGAACACAAGCCTACAGACTACAAGAAGGAGAATACAGGACCCCTGGAGACA 1380
 F  I  P  E  H  K  P  T  D  Y  K  K  E  N  T  G  P  L  E  T  460
    ACCCTA   AAC      GCA      AGGCAGCAGACT      CTC   CAGAAA
   (T) (S)   (N)      (A)     (R) (Q) (Q) (T)    (L)   (Q) (K)
   275 276   277      279     280 281 282 284    285   286 287
             TAC                  ---                     ATA
             (Y)                                          (I)
             278                  283                     288
```

275 - rs1570936011 – no publications, not in ClinVar – I442T
276 - rs754519752 – no publications, not in ClinVar – P443S
277 - rs758014823 – no publications, not in ClinVar – H445N
278 - rs758014823 – no publications, not in ClinVar – H445Y
279 - rs1274475754 – no publications, not in ClinVar – T448A
280 - rs1219574555 – no publications, not in ClinVar – K451R
281 - rs1472483040 – no publications, not in ClinVar – K452Q
282 - rs1272788889 – no publications, not in ClinVar – E453Q
283 - rs766334545 – no publications, not in ClinVar – E453del - in-frame deletion
284 - rs1296086963 – no publications, not in ClinVar – N454T
285 - rs558942258 – no publications, not in ClinVar – P457L
286 - rs1290015647 – no publications, not in ClinVar – E459Q
287 - rs746348181 – no publications, not in ClinVar – T460K
288 - rs746348181 – no publications, not in ClinVar – T460I

#ROW_NO.	INPUT	PROTEIN_ID	POSITION	RESIDUE_REF	RESIDUE_ALT	SCORE	PREDICTION (cutoff=-2.5)	#SEQ	#CLUSTER	SCORE	PREDICTION (cutoff=0.05)	MEDIAN_INFO	#SEQ
1	ENSP00000321345,442,I,T	ENSP00000321345	442	I	T	-0.44	Neutral	146	30	48	Damaging	3.44	27
2	ENSP00000321345,443,P,S	ENSP00000321345	443	P	S	-0.72	Neutral	146	30	105	Tolerated	3.44	27
3	ENSP00000321345,445,H,N	ENSP00000321345	445	H	N	-1.57	Neutral	146	30	26	Damaging	3.44	27
4	ENSP00000321345,445,H,Y	ENSP00000321345	445	H	Y	-0.80	Neutral	146	30	12	Damaging	3.44	27
5	ENSP00000321345,448,T,A	ENSP00000321345	448	T	A	-0.22	Neutral	146	30	125	Tolerated	3.33	26
6	ENSP00000321345,451,K,R	ENSP00000321345	451	K	R	-0.47	Neutral	146	30	408	Tolerated	3.33	25
7	ENSP00000321345,452,K,Q	ENSP00000321345	452	K	Q	-0.40	Neutral	146	30	65	Tolerated	3.33	25
8	ENSP00000321345,453,E,Q	ENSP00000321345	453	E	Q	-0.96	Neutral	146	30	35	Damaging	3.33	25
9	ENSP00000321345,454,N,T	ENSP00000321345	454	N	T	-0.49	Neutral	146	30	371	Tolerated	3.33	25
10	ENSP00000321345,457,P,L	ENSP00000321345	457	P	L	-0.46	Neutral	146	30	764	Tolerated	3.36	24
11	ENSP00000321345,459,E,Q	ENSP00000321345	459	E	Q	-0.75	Neutral	146	30	22	Damaging	3.34	25
12	ENSP00000321345,460,T,K	ENSP00000321345	460	T	K	-1.49	Neutral	146	30	73	Tolerated	3.33	26
13	ENSP00000321345,460,T,I	ENSP00000321345	460	T	I	-0.86	Neutral	146	30	27	Damaging	3.33	26

AGAGACTACCCGCAAAACTCGCTATTCGACAATACTACAGTTGTATATATTCCTGATCTC 1440
R D Y P Q N S L F D N T T V V Y I P D L 480

CAC	CTGCCA	TGGGTACTCAACAGTTCTATAGCT	CATTTT	GAAGTC
(H)	(L) (P)	(W) (V) (L) (N) (S) (S) (I) (A)	(H) (F)	(E) (V)
289	290 291	292 294 295 296 297 298 299 301	302 305	306 308
		TTG (L) 293		
		GCA (A) 300	GAT (D) 303	GGT (G) 307
			TGT (C) 304	

289 - rs775580555 – no publications, not in ClinVar – D462H
290 - rs138098976 – no publications, not in ClinVar – P464L
291 - rs1475068608 – no publications, not in ClinVar – Q465P
292 - rs772901651 – no publications, not in ClinVar – S467W
293 - rs772901651 – no publications, not in ClinVar – S467L
294 - rs759458205 – no publications, not in ClinVar – L468V
295 - rs1558269636 – no publications, not in ClinVar – F469L
296 - rs375054504 – no publications, not in ClinVar – D470N
297 - rs758022455 – no publications, not in ClinVar – N471S
298 - rs751235836 – no publications, not in ClinVar – T472S
299 - rs757737950 – no publications, not in ClinVar – T473I
300 - rs926027118 – no publications, not in ClinVar – T473A
301 - rs781688541 – no publications, not in ClinVar – V474A
302 - rs921734212 – no publications, not in ClinVar – Y476H
303 - rs921734212 – no publications, not in ClinVar – Y476D
304 - rs1272182280 – no publications, not in ClinVar – Y476C
305 - rs1159490612 – no publications, not in ClinVar – I477F
306 - rs369556867 – no publications, not in ClinVar – D479E
307 - rs746388939 – no publications, not in ClinVar – D479G
308 - rs761159118 – no publications, not in ClinVar – L480V

#ROW_NO.	INPUT	PROTEIN_ID	POSITION	RESIDUE_REF	RESIDUE_ALT	SCORE	PREDICTION (cutoff=-2.5)	#SEQ	#CLUSTER	SCORE	PREDICTION (cutoff=0.05)	MEDIAN_INFO	#SEQ
1	ENSP00000321345,462,D,H	ENSP00000321345	462	D	H	-0.86	Neutral	146	30	1	Damaging	3.33	26
2	ENSP00000321345,464,P,L	ENSP00000321345	464	P	L	-0.84	Neutral	146	30	130	Tolerated	3.36	24
3	ENSP00000321345,465,Q,P	ENSP00000321345	465	Q	P	-0.21	Neutral	146	30	265	Tolerated	3.33	26
4	ENSP00000321345,467,S,W	ENSP00000321345	467	S	W	-1.84	Neutral	146	30	9	Damaging	3.33	26
5	ENSP00000321345,467,S,L	ENSP00000321345	467	S	L	-1.70	Neutral	146	30	90	Tolerated	3.33	26
6	ENSP00000321345,468,L,V	ENSP00000321345	468	L	V	-0.27	Neutral	146	30	348	Tolerated	3.33	26
7	ENSP00000321345,469,F,L	ENSP00000321345	469	F	L	0.21	Neutral	146	30	1.000	Tolerated	3.33	26
8	ENSP00000321345,470,D,N	ENSP00000321345	470	D	N	-0.24	Neutral	146	30	171	Tolerated	3.33	26
9	ENSP00000321345,471,N,S	ENSP00000321345	471	N	S	0.35	Neutral	146	30	737	Tolerated	3.33	26
10	ENSP00000321345,472,T,S	ENSP00000321345	472	T	S	0.41	Neutral	146	30	807	Tolerated	3.33	27
11	ENSP00000321345,473,T,I	ENSP00000321345	473	T	I	-1.02	Neutral	146	30	5	Damaging	3.33	27
12	ENSP00000321345,473,T,A	ENSP00000321345	473	T	A	-1.03	Neutral	146	30	16	Damaging	3.33	27
13	ENSP00000321345,474,V,A	ENSP00000321345	474	V	A	-1.10	Neutral	146	30	39	Damaging	3.33	27
14	ENSP00000321345,476,Y,H	ENSP00000321345	476	Y	H	-1.92	Neutral	146	30	13	Damaging	3.33	27
15	ENSP00000321345,476,Y,D	ENSP00000321345	476	Y	D	-3.34	Deleterious	146	30	194	Tolerated	3.33	27
16	ENSP00000321345,476,Y,C	ENSP00000321345	476	Y	C	-3.06	Deleterious	146	30	11	Damaging	3.33	27
17	ENSP00000321345,477,I,F	ENSP00000321345	477	I	F	-0.99	Neutral	146	30	3	Damaging	3.33	27
18	ENSP00000321345,479,D,E	ENSP00000321345	479	D	E	-0.47	Neutral	146	30	20	Damaging	3.33	27
19	ENSP00000321345,479,D,G	ENSP00000321345	479	D	G	-1.55	Neutral	146	30	130	Tolerated	3.33	27
20	ENSP00000321345,480,L,V	ENSP00000321345	480	L	V	-0.37	Neutral	146	30	141	Tolerated	3.33	27

```
AACACTGGATATAAACCCCAAATTTCAAATTTTCTGCCTGAGGGAAGCCATCTCAGCAAT 1500
 N  T  G  Y  K  P  Q  I  S  N  F  L  P  E  G  S  H  L  S  N   500
    ATTAGA         CAC      CAT         AAGGAACGC      AAC
    (I)(R)         (H)      (H)         (K)(E)(R)      (N)
    309 311        313      315         317 318 319    321
    GCTGTA         CCA      GAT               GGC
    (A)(V)         (P)      (D)               (G)
    310 312        314      316               320
```

309 - rs1188184768 – no publications, not in ClinVar - T482I
310 - rs1237460156 – no publications, not in ClinVar – T482A
311 - rs1239064654 – no publications, not in ClinVar – G483R
312 - rs1421853018 – no publications, not in ClinVar – G483V
313 - rs139858085 – no publications, not in ClinVar - Q487H
314 - rs1164242345 – no publications, not in ClinVar - Q487P
315 - rs1459058332 – no publications, not in ClinVar – N490H
316 - rs1459058332 – no publications, not in ClinVar - N490D
317 - rs749335735 – no publications, not in ClinVar - E494K
318 - rs1208361755 – no publications, not in ClinVar – G495E
319 - rs768596081 – no publications, not in ClinVar - S496R
320 - rs768596081 – no publications, not in ClinVar – S496G
321 - rs1336720183 – no publications, not in ClinVar – S499N

#ROW_NO.	INPUT	PROTEIN_ID	POSITION	RESIDUE_REF	RESIDUE_ALT	SCORE	PREDICTION (cutoff=-2.5)	#SEQ	#CLUSTER	SCORE	PREDICTION (cutoff=0.05)	MEDIAN_INFO	#SEQ
1	ENSP00000321345,482,T,I	ENSP00000321345	482	T	I	-1.89	Neutral	146	30	20	Damaging	3.33	27
2	ENSP00000321345,482,T,A	ENSP00000321345	482	T	A	-0.92	Neutral	146	30	94	Tolerated	3.33	27
3	ENSP00000321345,483,G,R	ENSP00000321345	483	G	R	-2.52	Deleterious	146	30	0	Damaging	3.33	27
4	ENSP00000321345,487,Q,H	ENSP00000321345	487	Q	H	-1.52	Neutral	146	30	0	Damaging	3.33	27
5	ENSP00000321345,487,Q,P	ENSP00000321345	487	Q	P	-1.95	Neutral	146	30	0	Damaging	3.33	27
6	ENSP00000321345,490,N,H	ENSP00000321345	490	N	H	-0.74	Neutral	146	30	187	Tolerated	3.33	27
7	ENSP00000321345,490,N,D	ENSP00000321345	490	N	D	-1.04	Neutral	146	30	27	Damaging	3.33	27
8	ENSP00000321345,494,E,K	ENSP00000321345	494	E	K	-0.52	Neutral	146	30	150	Tolerated	3.32	26
9	ENSP00000321345,495,G,E	ENSP00000321345	495	G	E	-1.29	Neutral	146	30	106	Tolerated	3.32	26
10	ENSP00000321345,496,S,R	ENSP00000321345	496	S	R	-0.45	Neutral	146	30	15	Damaging	3.32	26
11	ENSP00000321345,496,S,G	ENSP00000321345	496	S	G	-0.64	Neutral	146	30	71	Tolerated	3.32	26
12	ENSP00000321345,499,S,N	ENSP00000321345	499	S	N	-0.14	Neutral	146	30	949	Tolerated	3.33	27

```
AATAATGAAATTACTTCCTTAACACTTAAACCACCAGTTGATTCCTTAGACTCAGGAAAT 1560
 N   N   E   I   T   S   L   T   L   K   P   P   V   D   S   L   D   S   G   N   520
    AGT             GCC     TCA     CAATCA          GGT     TCA     CCAAGA
    (S)             (A)     (S)     (Q) (S)         (G)     (S)     (P) (R)
    322             323     325     326 327         330     331     332 333
                    TTC               CAA                               GCA
                    (F)               (Q)                               (A)
                    324               328                               334
                                      CTA
                                      (L)
                                      329
```

322 - rs748364447 – no publications, not in ClinVar - N502S
323 - rs886133623 – no publications, not in ClinVar – S506A
324 - rs1283551780 – no publications, not in ClinVar – S506F
325 - rs1283342977 – no publications, not in ClinVar – T508S
326 - rs776354187 – no publications, not in ClinVar - K510Q
327 - rs759476112 – no publications, not in ClinVar - P511S
328 - rs1320708145 – no publications, not in ClinVar – P511Q
329 - rs1320708145 – no publications, not in ClinVar – P511L
330 - rs765181765 – no publications, not in ClinVar - D514G
331 - rs1570936478 – no publications, not in ClinVar - L516S
332 - rs373245679 – no publications, not in ClinVar - S518P
333 - rs530714168 – no publications, not in ClinVar – G519R
334 - rs763484544 – no publications, not in ClinVar – G519A

#ROW_NO.	INPUT	PROTEIN_ID	POSITION	RESIDUE_REF	RESIDUE_ALT	SCORE	PREDICTION (cutoff=-2.5)	#SEQ	#CLUSTER	SCORE	PREDICTION (cutoff=0.05)	MEDIAN_INFO	#SEQ
1	ENSP00000321345,502,N,S	ENSP00000321345	502	N	S	0.44	Neutral	146	30	266	Tolerated	3.33	27
2	ENSP00000321345,506,S,A	ENSP00000321345	506	S	A	-0.21	Neutral	146	30	771	Tolerated	3.33	27
3	ENSP00000321345,506,S,F	ENSP00000321345	506	S	F	-0.15	Neutral	146	30	49	Damaging	3.33	27
4	ENSP00000321345,508,T,S	ENSP00000321345	508	T	S	-0.27	Neutral	146	30	329	Tolerated	3.33	27
5	ENSP00000321345,510,K,Q	ENSP00000321345	510	K	Q	0.14	Neutral	146	30	137	Tolerated	3.33	27
6	ENSP00000321345,511,P,S	ENSP00000321345	511	P	S	-0.71	Neutral	146	30	196	Tolerated	3.31	26
7	ENSP00000321345,511,P,Q	ENSP00000321345	511	P	Q	-0.86	Neutral	146	30	18	Damaging	3.31	26
8	ENSP00000321345,511,P,L	ENSP00000321345	511	P	L	-1.15	Neutral	146	30	21	Damaging	3.31	26
9	ENSP00000321345,514,D,G	ENSP00000321345	514	D	G	-0.65	Neutral	146	30	60	Tolerated	3.49	25
10	ENSP00000321345,516,L,S	ENSP00000321345	516	L	S	-1.15	Neutral	146	30	94	Tolerated	3.33	26
11	ENSP00000321345,518,S,P	ENSP00000321345	518	S	P	-0.16	Neutral	146	30	387	Tolerated	3.34	23
12	ENSP00000321345,519,G,R	ENSP00000321345	519	G	R	-2.24	Neutral	146	30	0	Damaging	3.34	23
13	ENSP00000321345,519,G,A	ENSP00000321345	519	G	A	-1.98	Neutral	146	30	0	Damaging	3.34	23

AATCCCAGGTTA**CAAAAGCAT**CCT**AATTTTGCTTTT**TCTGTT**TCA**AGTGTGAATTCA**CTA** 1620

N P R L Q K H P N F A F S V S S V N S L 540

AAAAATCGT (K) (N) (R) 335 336 338

GATTGTACTTTA (D) (C) (T) (L) 339 340 341 343

GCA (A) 344

CCA (P) 345

AAC (N) 337

CCT (P) 342

335 - rs1252714660 – no publications, not in ClinVar – Q525K
336 - rs751326080 – no publications, not in ClinVar – K526N
337 - rs751326080 – no publications, not in ClinVar – K526N
338 - rs756910475 – no publications, not in ClinVar – H527R
339 - rs767996100 – no publications, not in ClinVar - N529D
340 - rs1466901710 – no publications, not in ClinVar – F530C
341 - rs112114661 – no publications, not in ClinVar – A531T
342 - rs112114661 – no publications, not in ClinVar – A531P
343 - rs1400483678 – no publications, not in ClinVar – F532L
344 - rs1451818608 – no publications, not in ClinVar - S535A
345 - rs1309165373 – no publications, not in ClinVar – L540P

#ROW_NO.	INPUT	PROTEIN_ID	POSITION	RESIDUE_REF	RESIDUE_ALT	SCORE	PREDICTION (cutoff=-2.5)	#SEQ	#CLUSTER	SCORE	PREDICTION (cutoff=0.05)	MEDIAN_INFO	#SEQ
1	ENSP00000321345,525,Q,K	ENSP00000321345	525	Q	K	0.87	Neutral	146	30	1.000	Tolerated	3.33	27
2	ENSP00000321345,526,K,N	ENSP00000321345	526	K	N	-0.22	Neutral	146	30	278	Tolerated	3.33	27
3	ENSP00000321345,527,H,R	ENSP00000321345	527	H	R	-1.07	Neutral	146	30	32	Damaging	3.33	27
4	ENSP00000321345,529,N,D	ENSP00000321345	529	N	D	-0.47	Neutral	146	30	335	Tolerated	3.33	27
5	ENSP00000321345,530,F,C	ENSP00000321345	530	F	C	-1.66	Neutral	146	30	38	Damaging	3.33	27
6	ENSP00000321345,531,A,T	ENSP00000321345	531	A	T	0.46	Neutral	146	30	213	Tolerated	3.33	27
7	ENSP00000321345,531,A,P	ENSP00000321345	531	A	P	-0.44	Neutral	146	30	27	Damaging	3.33	27
8	ENSP00000321345,532,F,L	ENSP00000321345	532	F	L	-1.34	Neutral	146	30	83	Tolerated	3.33	27
9	ENSP00000321345,535,S,A	ENSP00000321345	535	S	A	-0.74	Neutral	146	30	145	Tolerated	3.33	27
10	ENSP00000321345,540,L,P	ENSP00000321345	540	L	P	-1.19	Neutral	146	30	305	Tolerated	3.33	27

AGCAACACA**ATA**TTT**CTTGGA**GAATTA**AGC**CTC**ATATTA**AATCAA**GGA**GAA**TGCAGTTCT** 1680
S N T I F L G E L S L I L N Q G E C S S 560

ACA (T) 346; GTA (V) 347
CCTAGA (P) (R) 348 349
AAC (N) 350
ATGGTA (M) (V) 351 352
GAA (E) 353; GCA (A) 354
TTCCGTTTT (F) (R) (F) 355 358 359; AGC (S) 356; CGC (R) 357

346 - rs749727344 – no publications, not in ClinVar - I544T
347 - rs1350588209 – no publications, not in ClinVar - I544V
348 - rs1353483558 – no publications, not in ClinVar - L546P
349 - rs1570936596 – no publications, not in ClinVar - G547R
350 - rs1248727454 – no publications, not in ClinVar - S550N
351 - rs1334757949 – no publications, not in ClinVar - I552M
352 - rs778949002 – no publications, not in ClinVar - L553V
353 - rs550527020 – no publications, not in ClinVar - G556E
354 - rs550527020 – no publications, not in ClinVar - G556A
355 - rs1222525055 – no publications, not in ClinVar - C558F
356 - rs1442094471 – no publications, not in ClinVar – C558S
357 - rs1442094471 – no publications, not in ClinVar – C558R
358 - rs564574754 – no publications, not in ClinVar - S559R
359 - rs1210149461 – no publications, not in ClinVar - S560F

#ROW_NO.	INPUT	PROTEIN_ID	POSITION	RESIDUE_REF	RESIDUE_ALT	SCORE	PREDICTION (cutoff=-2.5)	#SEQ	#CLUSTER	SCORE	PREDICTION (cutoff=0.05)	MEDIAN_INFO	#SEQ
1	ENSP00000321345,544,I,T	ENSP00000321345	544	I	T	-1.06	Neutral	146	30	1	Damaging	3.33	27
2	ENSP00000321345,544,I,V	ENSP00000321345	544	I	V	-0.40	Neutral	146	30	6	Damaging	3.33	27
3	ENSP00000321345,546,L,P	ENSP00000321345	546	L	P	-1.81	Neutral	146	30	0	Damaging	3.33	27
4	ENSP00000321345,547,G,R	ENSP00000321345	547	G	R	-0.07	Neutral	146	30	9	Damaging	3.33	27
5	ENSP00000321345,550,S,N	ENSP00000321345	550	S	N	-1.02	Neutral	146	30	111	Tolerated	3.33	27
6	ENSP00000321345,552,I,M	ENSP00000321345	552	I	M	-0.60	Neutral	146	30	2	Damaging	3.33	27
7	ENSP00000321345,553,L,V	ENSP00000321345	553	L	V	-0.67	Neutral	146	30	5	Damaging	3.33	27
8	ENSP00000321345,556,G,E	ENSP00000321345	556	G	E	-1.89	Neutral	146	30	2	Damaging	3.33	27
9	ENSP00000321345,556,G,A	ENSP00000321345	556	G	A	-1.49	Neutral	146	30	13	Damaging	3.33	27
10	ENSP00000321345,558,C,F	ENSP00000321345	558	C	F	-0.49	Neutral	146	30	77	Tolerated	3.33	27
11	ENSP00000321345,558,C,S	ENSP00000321345	558	C	S	-0.10	Neutral	146	30	545	Tolerated	3.33	27
12	ENSP00000321345,558,C,R	ENSP00000321345	558	C	R	-0.35	Neutral	146	30	133	Tolerated	3.33	27
13	ENSP00000321345,559,S,R	ENSP00000321345	559	S	R	-0.88	Neutral	146	30	483	Tolerated	3.33	27
14	ENSP00000321345,560,S,F	ENSP00000321345	560	S	F	-1.34	Neutral	146	30	4	Damaging	3.32	26

```
CCTGACATACAAAACTCAGTAGAGGAGGAAACCACCATGCTTTTGGAAAATGATTCACCC 1740
 P  D  I  Q  N  S  V  E  E  E  T  T  M  L  L  E  N  D  S  P  580
       ATGAAA          ATAGGG    CAA       AAG
       (M)(K)          (I)(G)    (Q)       (K)
       360 361         363 364   365       366
          AGA                              GTG
          (R)                              (V)
          362                              367
```

```
360 - rs113943721     - no publications, not in ClinVar - I563M
361 - rs769458559     - no publications, not in ClinVar - Q564K
362 - rs775541608     - no publications, not in ClinVar - Q564R
363 - rs1231878015    - no publications, not in ClinVar - V567I
364 - rs1201280319    - no publications, not in ClinVar - E568G
365 - rs1344309440    - no publications, not in ClinVar - E570Q
366 - rs762798308     - no publications, not in ClinVar - M573K
367 - rs1298200002    - no publications, not in ClinVar - M573V
```

#ROW_NO.	INPUT	PROTEIN_ID	POSITION	RESIDUE_REF	RESIDUE_ALT	SCORE	PREDICTION (cutoff=-2.5)	#SEQ	#CLUSTER	SCORE	PREDICTION (cutoff=0.05)	MEDIAN_INFO	#SEQ
1	ENSP00000321345,563,I,M	ENSP00000321345	563	I	M	-0.51	Neutral	146	30	126	Tolerated	3.33	27
2	ENSP00000321345,564,Q,K	ENSP00000321345	564	Q	K	-0.65	Neutral	146	30	854	Tolerated	3.33	27
3	ENSP00000321345,564,Q,R	ENSP00000321345	564	Q	R	-0.46	Neutral	146	30	647	Tolerated	3.33	27
4	ENSP00000321345,567,V,I	ENSP00000321345	567	V	I	-0.20	Neutral	146	30	699	Tolerated	3.33	27
5	ENSP00000321345,568,E,G	ENSP00000321345	568	E	G	-1.98	Neutral	146	30	42	Damaging	3.33	27
6	ENSP00000321345,570,E,Q	ENSP00000321345	570	E	Q	-0.45	Neutral	146	30	9	Damaging	3.47	26
7	ENSP00000321345,573,M,K	ENSP00000321345	573	M	K	-1.44	Neutral	146	30	6	Damaging	3.33	27
8	ENSP00000321345,573,M,V	ENSP00000321345	573	M	V	-0.36	Neutral	146	30	289	Tolerated	3.33	27

```
AGTGAAACTATTCCAGAACAGACCCTGCTTCCTGATGAATTTGTCTCCTGTTTGGGGATC 1800
 S  E  T  I  P  E  Q  T  L  L  P  D  E  F  V  S  C  L  G  I  600
```

```
ACTAAA     ATCTCA          AACCGG   CTT    AAAATT        TCT
(T)(K)     (I)(S)          (N)(R)   (L)    (K)(I)        (S)
368 370    372 374         375 376  377    378 379       381
GGTGGA     ATG                                 TAT
(G)(G)     (M)                                 (Y)
369 371    373                                 380
```

368 - rs768272736 – no publications, not in ClinVar – S581T
369 - rs1169791532 – no publications, not in ClinVar – S581G
370 - rs898913808 – no publications, not in ClinVar – E582K
371 - rs1295997830 – no publications, not in ClinVar – E582G
372 - rs1395107049 – no publications, not in ClinVar - I584I
373 - rs1395107049 – no publications, not in ClinVar – I584M
374 - rs773929382 – no publications, not in ClinVar – P585S
375 - rs367746671 – no publications, not in ClinVar – T588N
376 - rs1436667845 – no publications, not in ClinVar – L589R
377 - rs1349677894 – no publications, not in ClinVar - P591L
378 - rs200877860 – no publications, not in ClinVar - E593K
379 - rs533280065 – no publications, not in ClinVar – F594I
380 - rs1432011679 – no publications, not in ClinVar - F594Y
381 - rs761140825 – no publications, not in ClinVar - C597S

#ROW_NO.	INPUT	PROTEIN_ID	POSITION	RESIDUE_REF	RESIDUE_ALT	SCORE	PREDICTION (cutoff=-2.5)	#SEQ	#CLUSTER	SCORE	PREDICTION (cutoff=0.05)	MEDIAN_INFO	#SEQ
1	ENSP00000321345,581,S,T	ENSP00000321345	581	S	T	-0.52	Neutral	146	30	71	Tolerated	3.33	27
2	ENSP00000321345,581,S,G	ENSP00000321345	581	S	G	0.36	Neutral	146	30	486	Tolerated	3.33	27
3	ENSP00000321345,582,E,K	ENSP00000321345	582	E	K	-0.97	Neutral	146	30	166	Tolerated	3.33	27
4	ENSP00000321345,582,E,G	ENSP00000321345	582	E	G	-1.21	Neutral	146	30	2	Damaging	3.33	27
5	ENSP00000321345,584,I,I	ENSP00000321345	584	I	I	0.00	Neutral	146	30	1.000	Tolerated	3.33	27
6	ENSP00000321345,584,I,M	ENSP00000321345	584	I	M	-0.77	Neutral	146	30	14	Damaging	3.33	27
7	ENSP00000321345,585,P,S	ENSP00000321345	585	P	S	-2.50	Deleterious	146	30	0	Damaging	3.33	27
8	ENSP00000321345,588,T,N	ENSP00000321345	588	T	N	-1.91	Neutral	146	30	55	Tolerated	3.33	27
9	ENSP00000321345,589,L,R	ENSP00000321345	589	L	R	-1.79	Neutral	146	30	1	Damaging	3.33	27
10	ENSP00000321345,591,P,L	ENSP00000321345	591	P	L	-2.33	Neutral	146	30	3	Damaging	3.33	27
11	ENSP00000321345,593,E,K	ENSP00000321345	593	E	K	-0.85	Neutral	146	30	15	Damaging	3.33	27
12	ENSP00000321345,594,F,I	ENSP00000321345	594	F	I	-0.78	Neutral	146	30	80	Tolerated	3.33	27
13	ENSP00000321345,594,F,Y	ENSP00000321345	594	F	Y	-0.72	Neutral	146	30	61	Tolerated	3.33	27
14	ENSP00000321345,597,C,S	ENSP00000321345	597	C	S	-3.22	Deleterious	146	30	0	Damaging	3.33	27

```
GTGAATGAGGAGTTGCCATCTATTAATACTTATTTTCCACAAAATATTTTGGAAAGCCAC 1860
 V  N  E  E  L  P  S  I  N  T  Y  F  P  Q  N  I  L  E  S  H  620
ATG       GAC          ACT   GCT          AAAAGTACTATG    AACCGC
(M)       (D)          (T)   (A)          (K) (S) (T) (M) (N) (R)
382       384          385   387          388 390 391 392 393 394
CTG                    GTT                CCA                 CAA
(L)                    (V)                (P)                 (Q)
383                    386                389                 395
```

```
382 - rs755379895    - no publications, not in ClinVar - V601M
383 - rs755379895    - no publications, not in ClinVar - V601L
384 - rs1250866602   - no publications, not in ClinVar - E604D
385 - rs752735535    - no publications, not in ClinVar - I608T
386 - rs960175040    - no publications, not in ClinVar - I608V
387 - rs1468346338   - no publications, not in ClinVar - T610A
388 - rs758642309    - no publications, not in ClinVar - Q614K
389 - rs982678126    - no publications, not in ClinVar - Q614P
390 - rs1267029527   - no publications, not in ClinVar - N615S
391 - rs1197056098   - no publications, not in ClinVar - I616T
392 - rs1170355212   - no publications, not in ClinVar - L617M
393 - rs747171538    - no publications, not in ClinVar - S619N
394 - rs200641667    - no publications, not in ClinVar - H620R
395 - rs967440017    - no publications, not in ClinVar - H620Q
```

#ROW_NO.	INPUT	PROTEIN_ID	POSITION	RESIDUE_REF	RESIDUE_ALT	SCORE	PREDICTION (cutoff=-2.5)	#SEQ	#CLUSTER	SCORE	PREDICTION (cutoff=0.05)	MEDIAN_INFO	#SEQ
1	ENSP00000321345,601,V,M	ENSP00000321345	601	V	M	-0.07	Neutral	146	30	329	Tolerated	3.33	27
2	ENSP00000321345,601,V,L	ENSP00000321345	601	V	L	-0.15	Neutral	146	30	1.000	Tolerated	3.33	27
3	ENSP00000321345,604,E,D	ENSP00000321345	604	E	D	-0.36	Neutral	146	30	425	Tolerated	3.33	27
4	ENSP00000321345,608,I,T	ENSP00000321345	608	I	T	-1.04	Neutral	146	30	94	Tolerated	3.33	27
5	ENSP00000321345,608,I,V	ENSP00000321345	608	I	V	-0.05	Neutral	146	30	188	Tolerated	3.33	27
6	ENSP00000321345,610,T,A	ENSP00000321345	610	T	A	0.08	Neutral	146	30	131	Tolerated	3.33	27
7	ENSP00000321345,614,Q,K	ENSP00000321345	614	Q	K	-1.39	Neutral	146	30	0	Damaging	3.33	27
8	ENSP00000321345,614,Q,P	ENSP00000321345	614	Q	P	-1.77	Neutral	146	30	0	Damaging	3.33	27
9	ENSP00000321345,615,N,S	ENSP00000321345	615	N	S	-1.00	Neutral	146	30	105	Tolerated	3.47	26
10	ENSP00000321345,616,I,T	ENSP00000321345	616	I	T	-0.80	Neutral	146	30	6	Damaging	3.47	26
11	ENSP00000321345,617,L,M	ENSP00000321345	617	L	M	-0.51	Neutral	146	30	28	Damaging	3.47	26
12	ENSP00000321345,619,S,N	ENSP00000321345	619	S	N	-0.78	Neutral	146	30	154	Tolerated	3.47	26
13	ENSP00000321345,620,H,R	ENSP00000321345	620	H	R	-1.00	Neutral	146	30	29	Damaging	3.47	26
14	ENSP00000321345,620,H,Q	ENSP00000321345	620	H	Q	-0.73	Neutral	146	30	79	Tolerated	3.47	26

TTC**AAT**AGGATT**TCACTCTTG**GAA**AAG**TAG	1890
F N R I S L L E K *	629

GAT **CCAGTCGTG** **ATG**
(D) **(P) (V) (V)** **(M)**
396 399 400 403 404

TAT **TTC**
(Y) **(F)**
397 401

AGT **CGC**
(S) **(R)**
398 402

396 - rs369367934 – no publications, not in ClinVar - N622D
397 - rs369367934 – no publications, not in ClinVar – N622Y
398 - rs1019572891 – no publications, not in ClinVar – N622S
399 - rs779703262 – no publications, not in ClinVar – S625P
400 - rs373028008 – no publications, not in ClinVar – L626V
401 - rs373028008 – no publications, not in ClinVar – L626F
402 - rs1234540068 – no publications, not in ClinVar – L626R
403 - rs1305260371 – no publications, not in ClinVar - L627V
404 - rs768672135 – no publications, not in ClinVar – K629M

#ROW_NO.	INPUT	PROTEIN_ID	POSITION	RESIDUE_REF	RESIDUE_ALT	SCORE	PREDICTION (cutoff=-2.5)	#SEQ	#CLUSTER	SCORE	PREDICTION (cutoff=0.05)	MEDIAN_INFO	#SEQ
1	ENSP00000321345,622,N,D	ENSP00000321345	622	N	D	-1.08	Neutral	146	30	32	Damaging	3.68	25
2	ENSP00000321345,622,N,Y	ENSP00000321345	622	N	Y	-0.75	Neutral	146	30	4	Damaging	3.68	25
3	ENSP00000321345,622,N,S	ENSP00000321345	622	N	S	-0.41	Neutral	146	30	436	Tolerated	3.68	25
4	ENSP00000321345,625,S,P	ENSP00000321345	625	S	P	-0.42	Neutral	146	30	100	Tolerated	3.68	25
5	ENSP00000321345,626,L,V	ENSP00000321345	626	L	V	-0.73	Neutral	146	30	0	Damaging	3.68	25
6	ENSP00000321345,626,L,F	ENSP00000321345	626	L	F	-1.68	Neutral	146	30	0	Damaging	3.68	25
7	ENSP00000321345,626,L,R	ENSP00000321345	626	L	R	-1.74	Neutral	146	30	0	Damaging	3.68	25
8	ENSP00000321345,627,L,V	ENSP00000321345	627	L	V	-0.72	Neutral	146	30	5	Damaging	3.68	25
9	ENSP00000321345,629,K,M	ENSP00000321345	629	K	M	-1.69	Neutral	146	30	1	Damaging	3.68	24

https://www.ncbi.nlm.nih.gov/snp

31190 SNPS in the IL-23R

404 annotated SNPs

398 missense
1 start codon (internal SNP number: 01)
3 in-frame deletions (internal SNP number: 56; 137; 283)
1 in-frame insertion (internal SNP number: 136)
1 missense stop codon (internal SNP number: 19)

8 missense with peer-reviewed publication

(internal SNP number: 03; 58; 93; 109; 111; 198; 231; 241)
